# Up-regulation of voltage-gated sodium channels by peptides mimicking S4-S5 linkers reveals a variation of the ligand-receptor mechanism

**DOI:** 10.1038/s41598-020-62615-6

**Published:** 2020-04-03

**Authors:** Olfat A. Malak, Fayal Abderemane-Ali, Yue Wei, Fabien C. Coyan, Gilyane Pontus, David Shaya, Céline Marionneau, Gildas Loussouarn

**Affiliations:** 1 Université de Nantes, CNRS, INSERM, l’institut du thorax, F-44000 Nantes, France; 2 Present Address: Buck Institute for Research on Aging, 8001 Redwood Blvd, Novato, California 94945 USA; 3 Present Address: Cardiovascular Research Institute, University of California, San Francisco, California 941158-9001 USA; 4 Present Address: Department of Cardiology, Shanghai Ruijin Hospital, Shanghai Jiao Tong University School of Medicine, Shanghai, China; 5 Cardiovascular Research Institute, University of California, San Francisco, California 941158-9001 USA

**Keywords:** Ion transport, Skeletal muscle

## Abstract

Prokaryotic Na_V_ channels are tetramers and eukaryotic Na_V_ channels consist of a single subunit containing four domains. Each monomer/domain contains six transmembrane segments (S1-S6), S1-S4 being the voltage-sensor domain and S5-S6 the pore domain. A crystal structure of Na_V_Ms, a prokaryotic Na_V_ channel, suggests that the S4-S5 linker (S4-S5_L_) interacts with the C-terminus of S6 (S6_T_) to stabilize the gate in the open state. However, in several voltage-gated potassium channels, using specific S4-S5_L_-mimicking peptides, we previously demonstrated that S4-S5_L_/S6_T_ interaction stabilizes the gate in the closed state. Here, we used the same strategy on another prokaryotic Na_V_ channel, Na_V_Sp1, to test whether equivalent peptides stabilize the channel in the open or closed state. A Na_V_Sp1-specific S4-S5_L_ peptide, containing the residues supposed to interact with S6_T_ according to the Na_V_Ms structure, induced both an increase in Na_V_Sp1 current density and a negative shift in the activation curve, consistent with S4-S5_L_ stabilizing the open state. Using this approach on a human Na_V_ channel, hNa_V_1.4, and testing 12 hNa_V_1.4 S4-S5_L_ peptides, we identified four activating S4-S5_L_ peptides. These results suggest that, in eukaryotic Na_V_ channels, the S4-S5_L_ of DI, DII and DIII domains allosterically modulate the activation gate and stabilize its open state.

## Introduction

Voltage-gated sodium channels (Na_V_) are crucial in excitable as well as non-excitable cells and mutations in Na_V_1.x-subunits have been associated with muscular, neuronal and cardiac channelopathies in human^1^. Voltage-gated potassium (K_V_) channels and prokaryotic Na_V_ channels are tetramers of subunits containing six transmembrane segments (S1 to S6). Each of the four subunits consists of one voltage-sensor domain (S1 to S4) and a pore domain (S5-S6). The four pore domains tetramerize to form a single pore module, which is regulated by the four voltage sensor domains. The arrangement of eukaryotic Na_V_ channels is similar, with one major difference: the channel is made of a single subunit containing four homologous domains, rather than four identical subunits. Each domain in eukaryotic Na_V_ channels is structurally equivalent to one subunit in K_V_ or prokaryotic Na_V_ channels, and consists of six transmembrane segments (S1 to S6).

Despite intensive work on the voltage-gating of K_V_ and Na_V_ channels, we still lack a clear picture describing the coupling between S4 voltage-sensor movement and S6 pore gating. Both structural and functional studies identified the linker between S4 and S5 (named S4-S5_L_) and the C-terminus of S6 (named S6_T_), as major actors in this coupling^2–21^. Different coupling mechanisms have been suggested. The crystal structure of K_V_1.2, and more recently the cryo-electron microscopy and crystal structures of both eukaryotic and prokaryotic Na_V_ channels suggested that the four S4-S5_L_ form a mechanical lever or a constriction ring intimately interacting with S6_T_ when the activation gate is closed. Upon membrane depolarization, constriction is relieved, and channel activation gate can open^10,12,15,19–21^. On the other hand, other studies performed on the bacterial Na_V_Ms (from Magnetococcus marinus) channel suggest that the S4-S5_L_ may also be involved in an interaction motif stabilizing the channel open state^8,16,17^. So rather than only playing the role of a constriction ring (obligatory role, as described for Shaker^22^), S4-S5_L_ may also allosterically modulate channel gating: the “up” or activated S4 conformation would favor but not impose the channel open state. Such allosteric regulation has been suggested for several channels, including hK_V_11.1 (hERG) and hK_V_7.1 (KCNQ1) channels^23,24^. In these channels, we elucidated the nature of this allosteric coupling: when S4 sensors are in the “down” or deactivated conformation, the four S4-S5_L_ bind to S6_T_ in the closed state, stabilizing this state^25,26^. Noteworthy, ATP has also been shown to stabilize the closed state of K_ATP_ channels^27,28^. In K_V_ channels, S4-S5_L_ can thus be seen as an inhibitor (like ATP) attached to the S4 voltage sensor. When the membrane is depolarized, S4 pulls S4-S5_L_ out of its binding pocket, leading to channel opening. This is consistent with the observation that specific S4-S5_L_-mimicking peptides inhibit hK_V_7.1 and hK_V_11.1 channels, by replacing the endogenous segment in the binding pocket^25,26^. This mechanism was recently extended to hK_V_10.2 channels^29^.

In the case of Na_V_ channels, such an allosteric model of the voltage-dependent gating mechanism has never been functionally tested. From the interaction motif observed in Na_V_Ms channel^8,16,17^, we hypothesized that S4-S5_L_ acts as a ligand binding to S6_T_ and stabilizing the channel open-state and not the closed state. We used the same peptide approach previously used for hK_V_7.1, hK_V_11.1 and hK_V_10.2 channels^25,26,29^ to test whether S4-S5_L_ peptides lead to a gain of function in both prokaryotic and eukaryotic Na_V_ channels.

We designed three S4-S5_L_ mimicking peptides specific for prokaryotic Na_V_Sp1 (from Silicibacter pomeroyi), and three S4-S5_L_ mimicking peptides specific for each of the four domains of hNa_V_1.4. None of these S4-S5_L_ peptides had an inhibitory effect. One S4-S5_L_ peptide from Na_V_Sp1 and at least one S4-S5_L_ peptide from DI, DII and DIII domains of hNa_V_1.4 promoted channel activity. Our results suggest that, as demonstrated in three K_V_ channels, the ligand/receptor model of interaction between S4-S5_L_ and S6_T_ applies also to both Na_V_Sp1 and hNa_V_1.4 channels, with one major difference: S4-S5_L_ stabilizes the open state in Na_V_ channels.

## Results

### A specific S4-S5_L_ mimicking peptide activates the bacterial channel Na_V_Sp1

**Figure 1:**
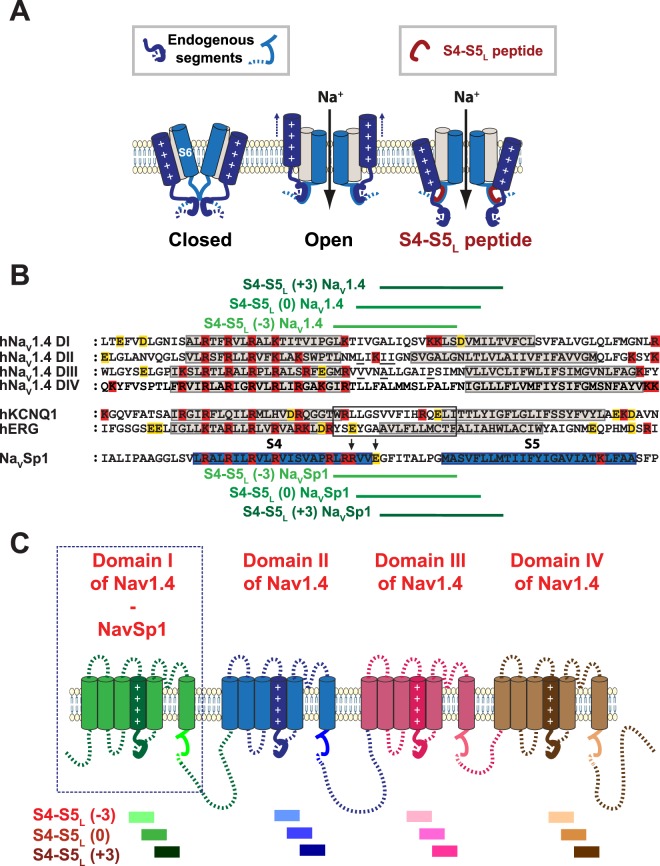
Ligand/receptor model. Multiple alignment used to design Na_V_Sp1 and Na_V_1.4 S4-S5_L_ peptides. (**A**) scheme of the ligand/receptor model in which S4-S5_L_ (endogenous segment, deep blue) binds to S6_T_ (endogenous segment, light blue) to stabilize the channel in the open state, as suggested by works on Na_V_Ms channel. The S4-S5_L_ peptide (red) mimics endogenous S4-S5_L_, stabilizing the channel open conformation. (**B)** Multiple alignment used to design Na_V_Sp1 and hNa_V_1.4 peptides from previously potent hK_V_7.1 and hK_V_11.1 S4-S5_L_ peptides (framed). Starting from S4-S5_L_(−3) peptide, two others peptides were designed, by shifting toward the C-terminus by 3 (S4-S5_L_(0)) and 6 amino acids (S4-S5_L_(+3)). Red: basic residues, yellow: acidic residues. Colored boxes represent the S4 and S5 segments. Mutated residues in skeletal channelopathies are underlined (in Na_V_1.4 S4-S5_L_). Arrows point to Na_V_Ms-corresponding residues interacting with S6_T_ (text) **C**: Scheme of the hNa_V_1.4 and Na_V_Sp1 channels showing the color used for each peptide/domain.

First, we tested the ligand/receptor model on Na_V_Sp1, a bacterial channel that is organized as a tetramer of identical subunits. If endogenous S4-S5_L_ acts like a ligand that stabilizes the activation gate in the open state, then a peptide mimicking endogenous S4-S5_L_ should increase Na_V_Sp1 channel activity (Fig. 1A). Three peptides were designed. One peptide, S4-S5_L_(−3), is aligned with the active peptides for hK_V_7.1^25^ and hK_V_11.1^26^ channel (Fig. 1B). Noteworthy, this peptide includes the sequence that aligns with Na_V_Ms RRVVQ motif. This RRVVQ motif engages a series of salt bridge and hydrogen-bonded interactions with S6_T_ and S3, such interactions playing a major role in channel open state stabilization^16^. Two other Na_V_Sp1 peptides, S4-S5_L_(0) and S4-S5_L_(+3) lack this sequence. Each Na_V_Sp1 peptide, S4-S5_L_(−3), S4-S5_L_(0) or S4-S5_L_(+3), was functionally tested separately. One peptide-encoding plasmid was co-transfected with the Na_V_Sp1-encoding plasmid. Results were compared to those from reference cells, co-transfected with Na_V_Sp1 and an unrelated peptide (hK_V_11.1 S6 C-terminal part, I663-T675, Control 1). An additional negative control, also unrelated to Na_V_ channels (hK_V_11.1 S4-S5_L_, A536-F551, Control 2) was used to confirm the absence of the Control 1 peptide effect. In all the following experiments, Control 2 did not show any significant difference, when compared to Control 1.

**Figure 2:**
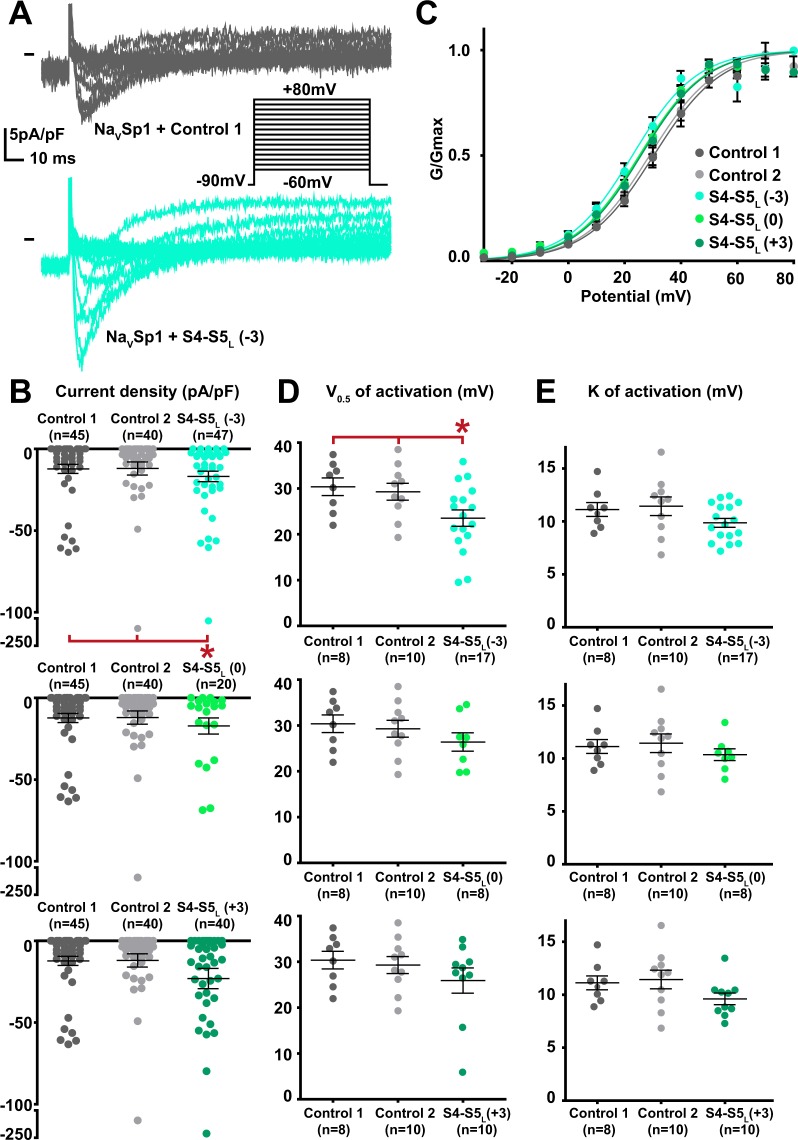
Effect of Na_V_Sp1 S4-S5_L_ mimicking peptides on Na_V_Sp1 current density and activation curve. (**A**) representative, superimposed current recordings in COS-7 cells transfected with Na_V_Sp1 and control 1 (top trace) or S4-S5_L_(−3) peptide (bottom trace). Inset: activation voltage protocol used (holding potential: −90 mV; 300-ms pulse at the indicated potentials; one sweep every 5 s). (**B)** Dot plot and mean ± sem of peak Na_V_Sp1 current densities recorded in COS-7 cells co-transfected with Na_V_Sp1 and the indicated peptide, at 30 mV. (**C)** Relative peak conductance *versus* membrane potential curves for Na_V_Sp1 channels in COS-7 cells co-transfected with Na_V_Sp1 and the indicated peptide. Lines are Boltzmann fits to the data. (**D,E)** Dot plot and mean ± sem of Na_V_Sp1 half-activation potential (V_0.5_; **D**) and activation slope (K; **E**) in COS-7 cells co-transfected with Na_V_Sp1 and the indicated peptide. *p value *vs*. both controls <0.05.

When co-expressed with Na_V_Sp1, the S4-S5_L_(−3) peptide provoked a gain of function on the current density (Fig. 2, Supplemental Table 1). Moreover, the activation curve was shifted to more negative potential with no concurrent shift of the voltage-dependence of the activation/inactivation kinetics. This latter observation excludes a membrane charge screening, locally changing the potential detected by the voltage-sensor (Supplemental Fig. 1). It is possible that enhancement of the Na_V_Sp1 current density of Na_V_Sp1 at 30 mV stimuli is partially caused by the negative shift of voltage dependent activation. Since a gain of function may also lead to incomplete channel deactivation^25^, we also tested if the current measured at −90mV, in the presence of S4-S5_L_ peptides, was greater than in ctrl1 and ctrl2 conditions. Our data shows that this is not the case (Supplemental Fig. 2), suggesting that channel deactivation is complete in the presence of peptides. The other two peptides, S4-S5_L_(0) and S4-S5_L_(+3) lacking the sequence aligning to the Na_V_Ms RRVVQ motif had no effect. The gain of function, caused by Na_V_Sp1 S4-S5_L_(−3) peptide suggests that Na_V_Sp1 follows a ligand/receptor model of voltage-dependent gating, with S4-S5_L_ stabilizing the channel in the open state.

**Figure 3:**
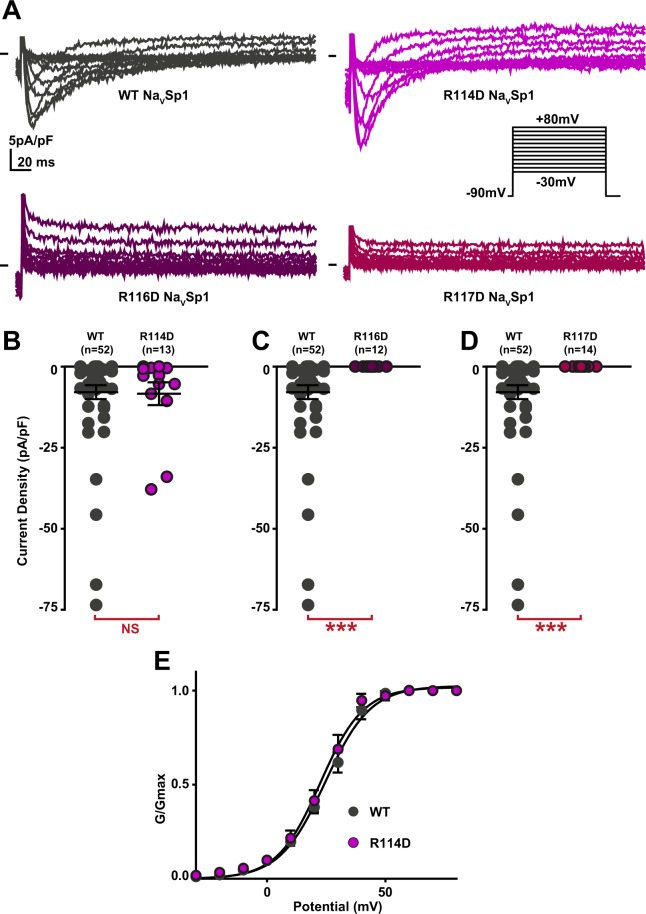
Effect of charge reversal in amino acids present in Na_V_Sp1 S4-S5_L_(−3) activating peptide on Na_V_Sp1 current density and activation curve. (**A**) representative, superimposed recordings of WT and mutant Na_V_Sp1 current. Activation voltage protocol used is the same as in Fig. 2. (**B–D)** Dot plot and mean ± sem of peak current densities recorded in COS-7 cells transfected with WT or mutant Na_V_Sp1, at 30 mV. (**E)** Relative peak conductance *versus* membrane potential curves for WT or mutant Na_V_Sp1 channels transfected in COS-7. Lines are Boltzmann fits to the data. ***p value *vs*. WT < 0.001.

**Figure 4:**
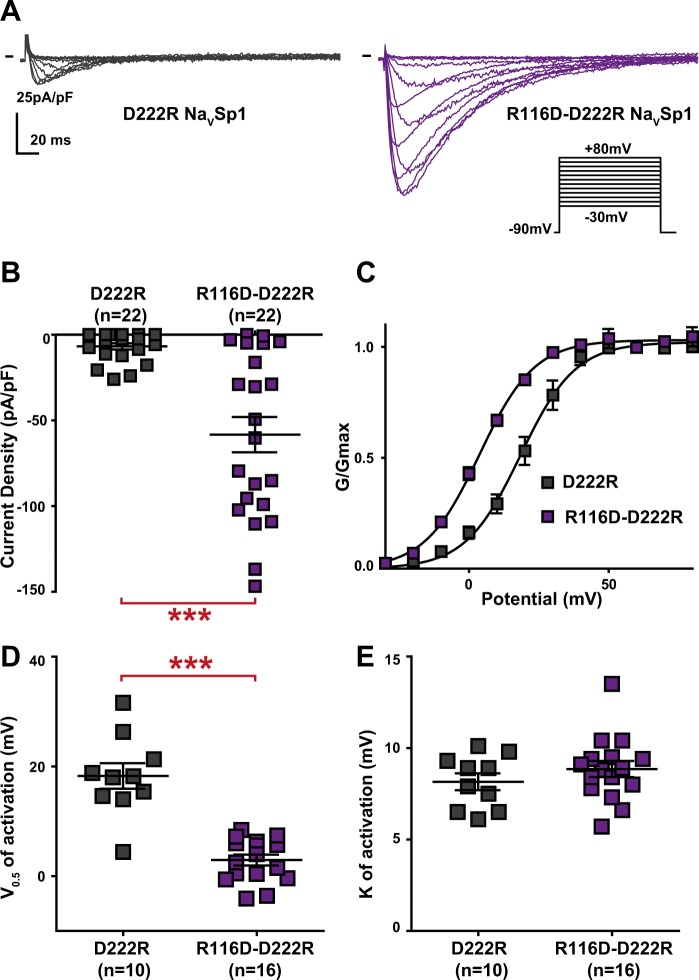
Opposite charges at position 116 (Na_V_Sp1 S4-S5_L_) and 222 (Na_V_Sp1 S6_T_) stabilizes the Na_V_Sp1 channel open state. (**A**) representative, superimposed current recordings of single Na_V_Sp1 mutant D222R and double mutant D222R/R116D. Activation voltage protocol used is the same as in Fig. 2. (**B)** Dot plot and mean ± sem of peak current densities recorded in COS-7 cells transfected with D222R or D222R/R116D Na_V_Sp1, at 30 mV. (**C)** Relative peak conductance *versus* membrane potential curves for D222R or D222R/R116D Na_V_Sp1 channels transfected in COS-7 cells. Lines are Boltzmann fits to the data. (**D**,**E)** Dot plot and mean ± sem of Na_V_Sp1 half-activation potential (V_0.5_; **D**) and activation slope (K; **E**) in COS-7 cells transfected with D222R or D222R/R116D Na_V_Sp1. ***p value *vs*. D222R < 0.001.

Because the active peptide S4-S5_L_(−3) contains two arginines (R116 and R117) that are absent in the inactive peptides (S4-S5_L_(0) and S4-S5_L_(+3)), we tested the role of these positively charged residues in Na_V_Sp1 gating. Charged amino-acid distribution in S4-S5_L_ and S6_T_ is quite different between Na_V_Sp1 and Na_V_Ms, with an additional arginine at the start of Na_V_Sp1 S4-S5_L_ (R114, before R116 and R117, cf. black arrows pointing to R in Supplemental Fig. 3) and an additional aspartate at position 222 in S6_T_ (black arrow pointing to D in Supplemental Fig. 3). In order to test the potential contribution of R114, R116 and R117 to the stabilization of the open state, we mutated one by one each arginine to an aspartate carrying the opposite charge. R114D did not have any effect on channel activity (Fig. 3A–B,E), but both R116D and R117D led to nonfunctional channels (Fig. 3A,C–D), consistent with a major role of both arginines in Na_V_Sp1 open state stabilization by both the endogenous and exogenous peptides. But because of the absence of detectable current, we could not exclude that R116D and R117D were preventing channel trafficking to the membrane. In order to confirm electrostatic interaction between S4-S5_L_ and S6_T_ to stabilize Na_V_Sp1 open state, we had to identify residues in S6_T_ with which R116 and/or R117 interacts. D222 was, among other residues, a good candidate because it is in a region that aligns with Na_V_Ms residues interacting with S4-S5_L_ to stabilize the channel open state (Supplemental Fig. 3). Interestingly, the addition of the D222R mutation on top of the nonfunctional R116D mutant was not only able to restore channel activity but also led to a channel that is more prone to be open, as compared to when both amino acids at position 116 and 222 carry a positive charge (D222R): we observed an increase in current amplitude and a −20-mV shift in the activation curve (Fig. 4). Such activity restoration and gain of function when amino acids at position 116 and 222 carry opposite charges (R116D + D222R) suggest that both endogenous and exogenous S4-S5_L_ peptides stabilize the channel open state through specific S4-S5_L_ and S6_T_ interaction.

### hNa_V_1.4 S4-S5_L_ peptides activate hNa_V_1.4

We also tested the ligand/receptor model on the hNa_V_1.4 voltage-gated channel that is organized as a single subunit of four homologous domains^11,15,20,21^. Again, three S4-S5_L_-encoding plasmids were designed for each domain, based on sequence alignment with hK_V_7.1 and hK_V_11.1 (Fig. 1B). Each of the 12 designed S4-S5_L_ peptides was tested separately: each hNa_V_1.4 S4-S5_L_ peptide-encoding plasmid was co-transfected with hNa_V_1.4 and hNa_V_β1-encoding plasmids.

**Figure 5:**
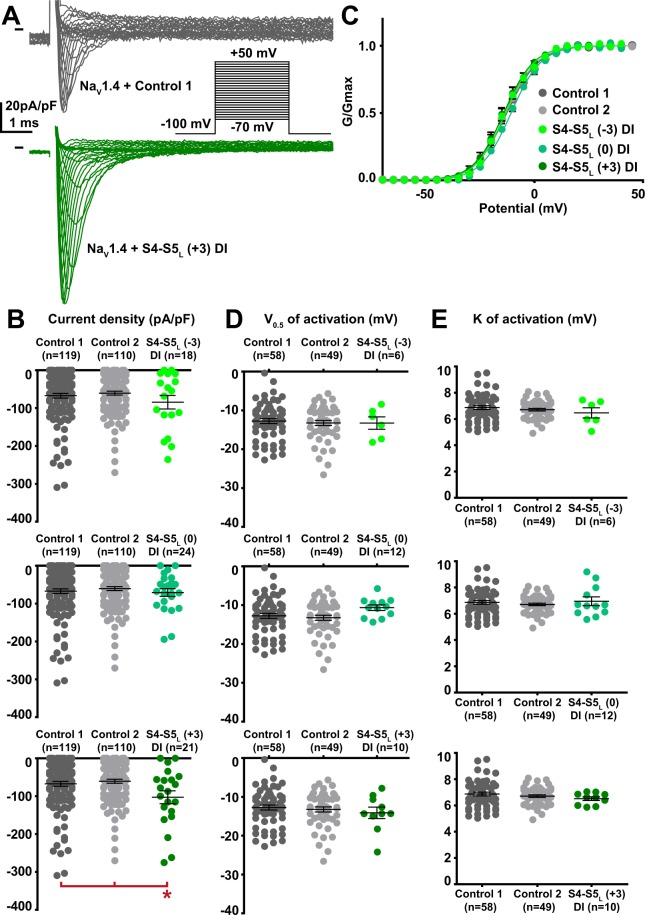
Effect of Na_V_1.4 S4-S5_L_ mimicking peptides of domain I on Na_V_1.4 current density and activation curve. (**A**) representative, superimposed current recordings in COS-7 cells co-transfected with Na_V_1.4, Na_V_ß1, and control 1 (top trace) or domain I S4-S5_L_(+3) peptide (bottom trace). Inset: activation voltage protocol used (holding potential: −100 mV; 30-ms pulse; one sweep every 2 s). (**B)** Dot plot and mean ± sem of peak Na_V_1.4 current densities recorded in COS-7 cells co-transfected with Na_V_1.4, Na_V_ß1, and the indicated peptide, at 0 mV. **C:** Relative peak conductance *versus* membrane potential curves for Na_V_1.4 channels in the same cell groups as in (**B)**. Lines are Boltzmann fits to the data. (**D**,**E)** Dot plot and mean ± sem of Na_V_1.4 half-activation potential (V_0.5_; **D**) and activation slope (K; **E**) in the same cells group as in (**B)**. *p value *vs*. both controls <0.05.

**Figure 6:**
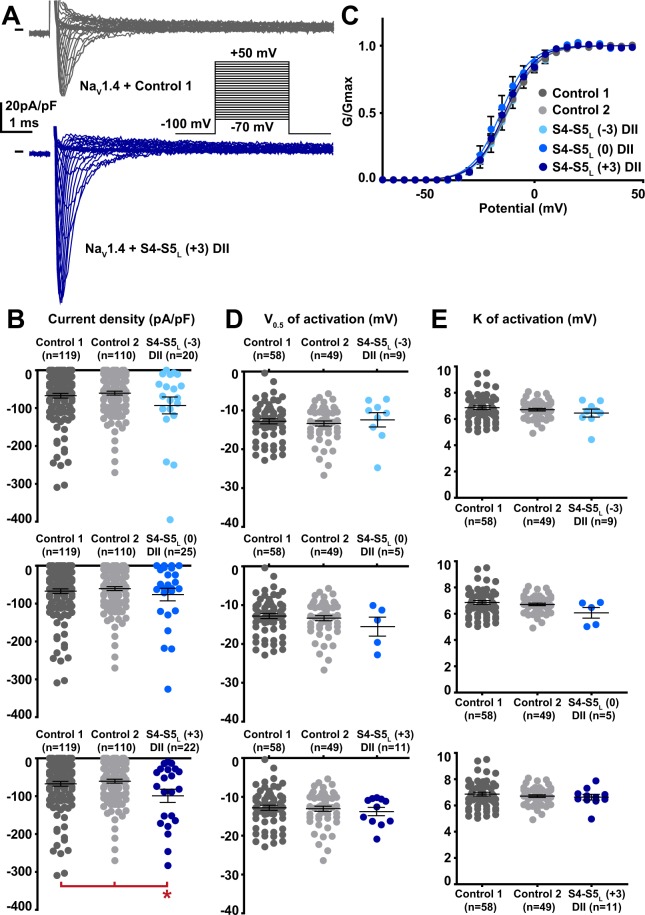
Effect of Na_V_1.4 S4-S5_L_ mimicking peptides of domain II on Na_V_1.4 current density and activation curve. (**A**) representative, superimposed current recordings in COS-7 cells co-transfected with Na_V_1.4, Na_V_ß1, and control 1 (top trace) or domain II S4-S5_L_(+3) peptide (bottom trace). Inset: activation voltage protocol used (holding potential: −100 mV; 30-ms pulse; one sweep every 2 s). (**B)** Dot plot and mean ± sem of peak Na_V_1.4 current densities recorded in COS-7 cells co-transfected with Na_V_1.4, Na_V_ß1, and the indicated peptide, at 0 mV. (**C**) Relative peak conductance *versus* membrane potential curves for Na_V_1.4 channels in the same cell groups as in (**B)**. Lines are Boltzmann fits to the data. (**D**,**E)** Dot plot and mean ± sem of Na_V_1.4 half-activation potential (V_0.5_; **D**) and activation slope (K; **E**) in the same cells group as in (**B)**. *p value *vs*. both controls <0.05.

**Figure 7:**
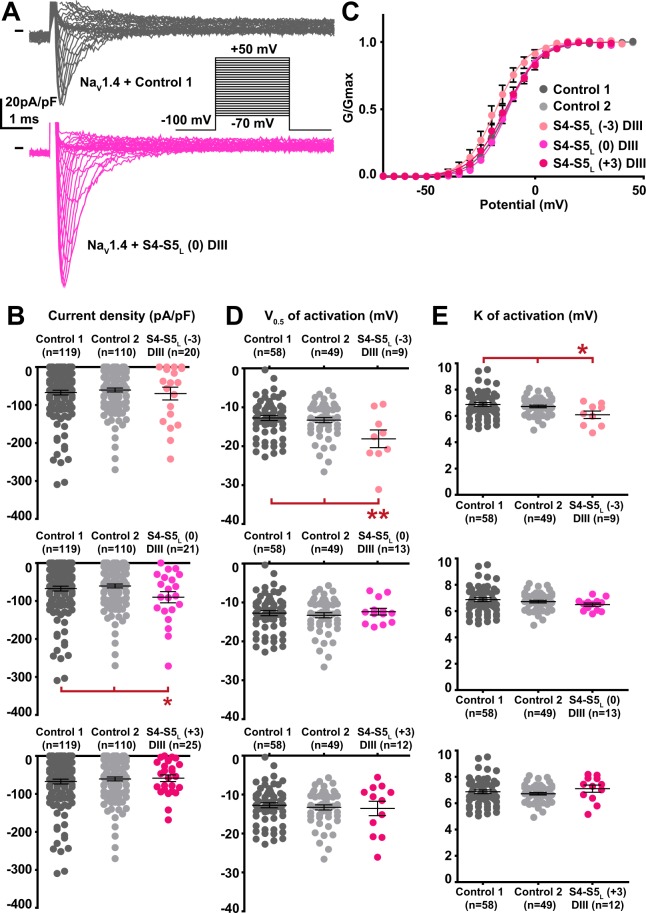
Effect of Na_V_1.4 S4-S5_L_ mimicking peptides of domain III on Na_V_1.4 current density and activation curve. (**A**) representative, superimposed current recordings in COS-7 cells co-transfected with Na_V_1.4, Na_V_ß1, and control 1 (top trace) or domain III S4-S5_L_(0) peptide (bottom trace). Inset: activation voltage protocol used (holding potential: −100 mV; 30-ms pulse; one sweep every 2 s). (**B)** Dot plot and mean ± sem of peak Na_V_1.4 current densities recorded in COS-7 cells co-transfected with Na_V_1.4, Na_V_ß1, and the indicated peptide, at 0 mV. **C:** Relative peak conductance *versus* membrane potential curves for Na_V_1.4 channels in the same cell groups as in (**B)**. Lines are Boltzmann fits to the data. (**D**,**E)** Dot plot and mean ± sem of Na_V_1.4 half-activation potential (V_0.5_; **D**) and activation slope (K; **E**) in the same cells group as in (**B)**. *p value *vs*. both controls <0.05. **p value *vs*. both controls <0.01.

**Figure 8:**
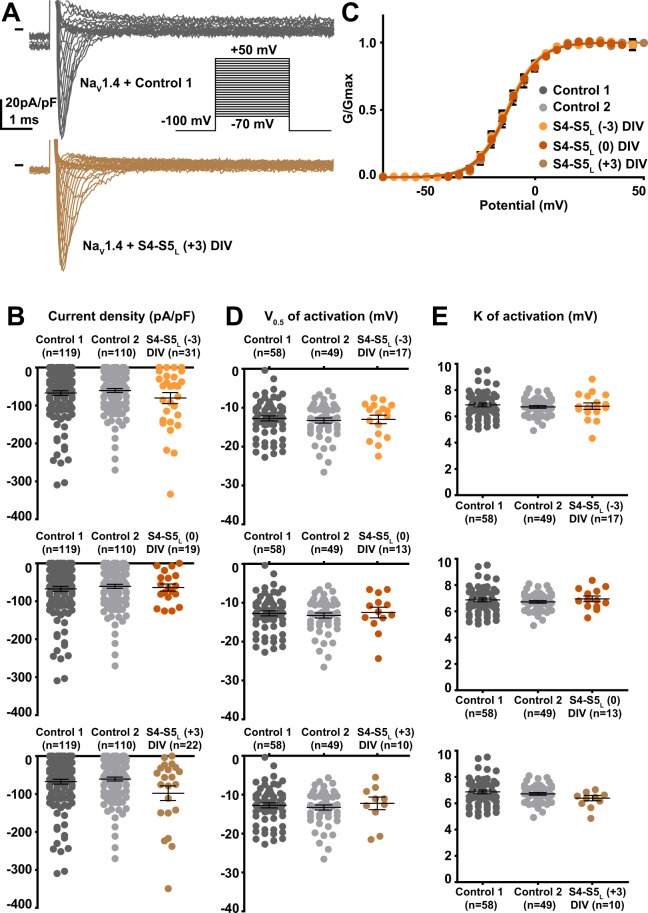
Effect of Na_V_1.4 S4-S5_L_ mimicking peptides of domain IV on Na_V_1.4 current density and activation curve. (**A**) representative, superimposed current recordings in COS-7 cells co-transfected with Na_V_1.4, Na_V_ß1, and control 1 (top trace) or domain IV S4-S5_L_(+3) peptide (bottom trace). Inset: activation voltage protocol used (holding potential: −100 mV; 30-ms pulse; one sweep every 2 s). (**B**) Dot plot and mean ± sem of peak Na_V_1.4 current densities recorded in COS-7 cells co-transfected with Na_V_1.4, Na_V_ß1, and the indicated peptide, at 0 mV. (**C**) Relative peak conductance *versus* membrane potential curves for Na_V_1.4 channels in the same cell groups as in (**B**). Lines are Boltzmann fits to the data. (**D**,**E**) Dot plot and mean ± sem of Na_V_1.4 half-activation potential (V_0.5_; **D**) and activation slope (K; **E**) in the same cells group as in (**B**).

Among the 12 tested hNa_V_1.4 S4-S5_L_ peptides, three peptides increased the hNa_V_1.4 current density. These activating peptides mimic three of the four hNa_V_1.4 S4-S5 linkers, in domain I (S4-S5_L_(+3)), domain II (S4-S5_L_(+3)) and domain III (S4-S5_L_(0)) of hNa_V_1.4 (Figs. 5B–8B, Supplemental Table 2).

One additional peptide in domain III shifted the activation curve to more negative potentials (S4-S5_L_(−3), Supplemental Table 2; Fig. 7C–E), also leading to a gain of function. This S4-S5_L_(−3) peptide is different from the S4-S5_L_(0) peptide that increased the current density in the same domain: it is shifted by three amino acids toward the N-terminus. We did not observe any alteration of the activation/inactivation kinetics by any of the peptides (Supplemental Fig. 4).

### S4-S5_L_ peptides do not modify hNa_V_1.4 channel trafficking

**Figure 9:**
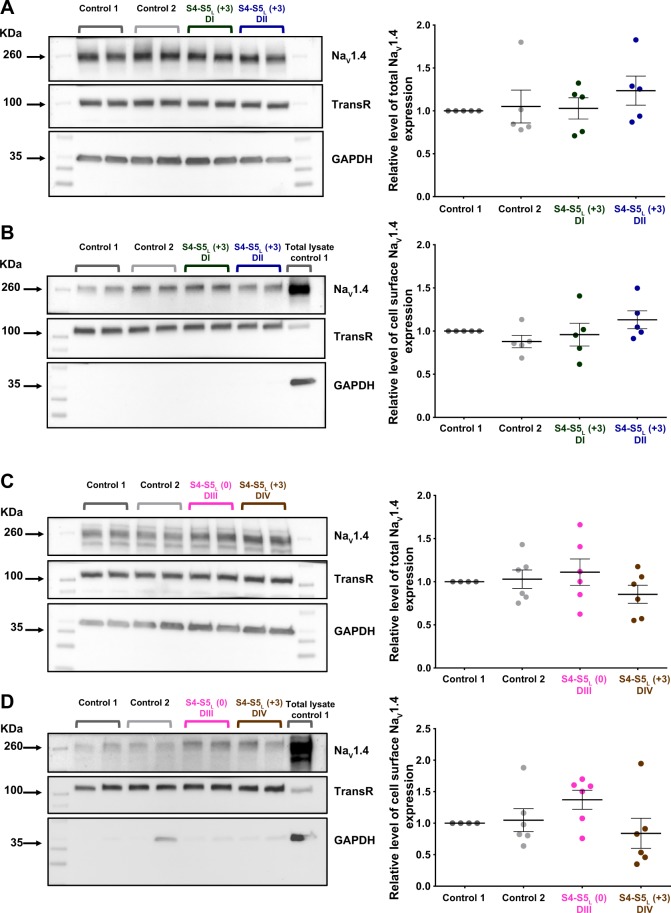
Effect on Na_V_1.4 channel expression of S4-S5_L_ mimicking peptides associated with an increased current density. Left: (**A**,**C)** representative western blots of total Na_V_1.4, transferrin receptor (TransR) and GAPDH from transfected COS-7 cells, in the presence of various control and S4-S5_L_ peptides as indicated. (**B**,**D)** representative western blots of the cell surface fraction of Na_V_1.4, transferrin receptor (TransR) and GAPDH from transfected COS-7 cells, in the presence of various control and S4-S5_L_ peptides. Right: corresponding quantifications of normalized mean ± sem intensities. Band intensities are first normalized to the intensity of the corresponding TransR bands, and ratios are then normalized to control 1 condition. In all condition, p > 0.05. In A–D, the three blots, realized on the same membrane, are cropped. Full-length blots of each tested protein are reported in Supplemental Fig. 5.

Cell surface biotinylation experiments were performed in order to verify whether increased current densities were due to gating alteration or to an increased channel trafficking. These experiments were done using the three peptides in domain I, II, III of hNa_V_1.4 that were causing an increase in current density, and using the S4-S5_L_(+3) peptide in domain IV of hNa_V_1.4 that was showing a trend of increased current density, although not significant. Neither the total nor the biotinylated fraction (plasma membrane) of hNa_V_1.4 protein was increased by any of the peptides, suggesting that domain I S4-S5_L_(+3), domain II S4-S5_L_(+3) and domain III S4-S5_L_(0) peptides increase hNa_V_1.4 current density through an alteration of channel gating and not its trafficking (Fig. 9; Supplemental Fig. 5).

Thus, out of the 12 tested peptides, four led to a gain of function of hNa_V_1.4 channel through an effect on channel gating.

### Effects of combination of peptides

**Figure 10:**
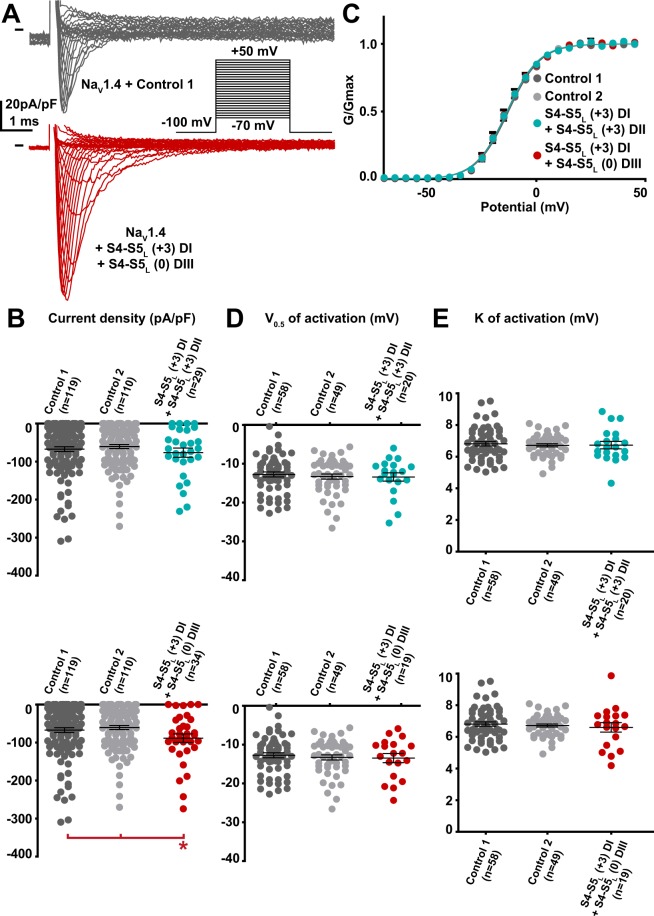
Effect of combination of two Na_V_1.4 S4-S5_L_ mimicking peptides that both had an effect on Na_V_1.4 current density when expressed alone. (**A**) representative, superimposed current recordings in COS-7 cells co-transfected with Na_V_1.4, Na_V_ß1, and control 1 (top trace) or the combination of domain I S4-S5_L_(+3) peptide and domain III S4-S5_L_(0) peptide (bottom trace). Inset: activation voltage protocol used (holding potential: −100 mV; 30-ms pulse; one sweep every 2 s). (**B)** Dot plot and mean ± sem of peak Na_V_1.4 current densities recorded in COS-7 cells co-transfected with Na_V_1.4, Na_V_ß1, and the indicated peptides, at 0 mV. **C**: Relative peak conductance *versus* membrane potential curves for Na_V_1.4 channels in the same cell groups as in (**B)**. Lines are Boltzmann fits to the data. (**D**,**E**) Dot plot and mean ± sem of Na_V_1.4 half-activation potential (V_0.5_; **D**) and activation slope (K; **E**) in the same cells group as in (**B)**. *p value *vs*. both controls <0.05.

Since in hNa_V_1.4 the S4-S5 linker sequences of the four domains differ, we explored if co-transfecting two activating peptides exerts a stronger effect on hNa_V_1.4 current density than the individual peptides, as two domains will be stabilized open instead of only one. In order to keep the same expression level of the channel, we needed to keep the same total DNA quantity in all conditions. Thus, to combine two peptides we added half quantity of each peptide-encoding plasmid, as compared to conditions with only one peptide. We did not observe any increase in current density when DI-S4-S5_L_(+3) and DII-S4-S5_L_(+3) peptides were co-expressed. This observation suggests that combination of the two peptides in lesser quantity was not as potent as when only one peptide was expressed (Fig. 10). It is possible that the presence of (i) smaller quantity of peptides in addition to (ii) some steric hindrance prevent the activating effect. Noteworthy, domains I and II are adjacent, consistent with the hypothetical steric hindrance. To limit the effect of steric hindrance, we selected activating peptides from two non-adjacent domains, namely DI-S4-S5_L_(+3) and DIII-S4-S5_L_(0). Indeed, co-expression of these DI-S4-S5_L_(+3) and DIII-S4-S5_L_(0) peptides caused an increase in the hNa_V_1.4 current density. Such an increase was similar but not greater than when only one peptide was expressed, probably because each of the peptides was present in lesser quantity. This observation highlights a limit of the model in which S4-S5 effects are not strong enough to potentially quantify the synergistic effect of the combination of peptides.

### S4-S5_L_ peptides modify hNa_V_1.4 channel inactivation

Since mutations in domains I, III and IV S4-S5_L_ have been associated with a large modification of the Na_V_1.4 channel fast inactivation^30–32^, we also tested the effect of the peptides on channel inactivation. We observed an increase in the slope factor of the inactivation curve when DI-S4-S5_L_(+3) or DIII-S4-S5_L_(+3) peptide was expressed (Supplemental Figs. 6–9; Supplemental Table 2). Also, and consistent with the effect of the combination of peptides on the activation curve, effect of DI-S4-S5_L_(+3) was still observed when it was co-expressed with the peptide corresponding to the non-adjacent domain (III), but not with the peptide corresponding to the adjacent domain (II) (Supplemental Fig. 10).

## Discussion

**Figure 11:**
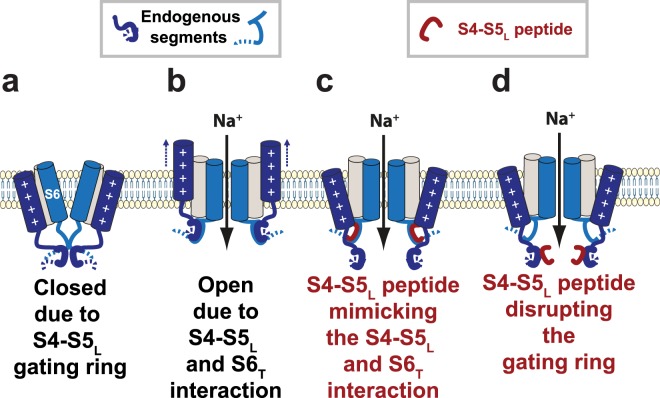
Summarizing schemes. (**a,b**) Two mechanisms of voltage-dependent gating. (**a**) The gating ring model in which the four S4-S5_L_ (endogenous segments, deep blue, only two are shown) constitute a constriction ring preventing S6 (endogenous segment, light blue) iris-like dilation. (**b**) Scheme of the ligand/receptor model in which S4-S5_L_ binds to S6_T_ to stabilize the channel in the open state. (**c)** The S4-S5_L_ peptides (red) mimic the effect of endogenous S4-S5_L_ described in (b), stabilizing the open conformation. (**d**) Alternatively, the S4-S5_L_ peptides (red), interact with endogenous S4-S5_L_, destabilizing the gating ring described in (a) and hence lead to channel opening.

In this work, we used a S4-S5_L_ mimicking peptide approach to test whether voltage-gated sodium channels follow the ligand/receptor model previously proposed for hK_V_7.1^25^, hK_V_11.1^26^ and hK_V_10.2^29^ channels. We identified one activating S4-S5_L_ peptide in Na_V_Sp1 and four in hNa_V_1.4, suggesting that Na_V_ channels follow a ligand/receptor model of voltage-dependent gating (Fig. 11c): when the membrane is depolarized, endogenous S4-S5_L_ stabilizes the open state of Na_V_ channels, as indicated by the Na_V_Ms structure captured in the open state^8,16,17^. This contrasts with what is happening with K_V_ channels: when the membrane is polarized, endogenous S4-S5_L_ stabilizes the closed state of K_V_ channels, as suggested by several studies^25,26,29^.

The various peptide effects, either on current density or on the activation voltage dependence, suggest that peptides are acting on different conformational transitions leading to channel opening. Due to the multi-state process of channel voltage-dependent gating, implying several conformational changes, peptides affinity may be high enough for the alteration of one parameter (e.g., current density), but not for the alteration of the other one (e.g., V_0.5_, slope factor). The peptides effects on current density but not on the activation voltage dependence have already been observed in hK_V_11.1 channel^26^ and hK_V_7.1 channels^25^ and were also described in a kinetic model of the peptide effect on K_V_10.2 channels^29^. In hK_V_11.1 channel, we show that a S6_T_ mimicking peptide has only an effect on the current density when affinity is low but can also drastically change the activation curve when its affinity is increased by a specific disulfide bridge^26^.

Here, all the data obtained on hNa_V_1.4 suggest that S4-S5_L_ in domain I, II and III play a significant role in the channel voltage dependence of activation. In the neuronal channel Na_V_1.2, mutations of S4 gating charges in all four domains were found to affect the activation^33^. Nevertheless, of the mutations that shifted the V_0.5_ of activation, the most pronounced effects were observed when the fourth charge in each of domains I, II, and III was neutralized. This suggests that domains I to III play a critical role in coupling the voltage sensor with the activation gate of Na_V_1.2, consistent with our results on hNa_V_1.4. Moreover, voltage-clamp fluorimetry experiments performed on Na_V_1.4 S4 segments showed that domain I, II and III play a significant role in the channel voltage-dependence of activation, also consistent with our results on hNa_V_1.4^34^.

Although consistent with a ligand/receptor model, S4-S5_L_ peptides effects on Na_V_Sp1 and hNa_V_1.4 are moderate. It is worth mentioning that these effects are nevertheless in the range of those observed in previous studies on three different voltage-gated potassium channels^25,26,29^. In the previous studies, we interpreted that the S4-S5_L_/S6_T_ interaction has to be loose, probably due to the low affinity between native S4-S5_L_ and S6_T_, which is necessary for S4-S5_L_ ligand unbinding and channel opening during membrane depolarization^26^. In the channel, this low affinity is compensated by the imposed proximity of the two segments. Experimentally, this can be compensated only partly by high peptide concentration. In two K_V_ channels, we found a way to reinforce S4-S5_L_ peptide binding to S6_T_ via a specific disulfide bridge between two cysteines, and hence, increase their effects on the channel^26,29^. It would be interesting to identify such a pair of cysteines in Na_V_ channels.

Because of the moderate peptides effects, we cannot be sure that the ligand/receptor is the major actor of signal transduction between S4 movement and pore opening in Na_V_ channels. Other mechanisms such as the constriction ring mechanism, suggested by several Na_V_ channels structures, certainly play a major role^10,12,15,20,21^. The S4-S5_L_ peptides may also disrupt the constriction ring and by this way lead to channel opening (Fig. 11d). But the fact that Na_V_Sp1 most potent peptides contain amino-acids that play a major role in channel open state stabilization (Figs. 3 and 4), strongly supports the hypothesis that these peptides (endogenous or exogenous peptides) bind to the C-terminal end of S6 and stabilize the channel open state (Fig. 11c).

Both mechanisms (Constriction ring in Fig. 11a and ligand-receptor in Fig. 11b) may coexist: the interaction between S4-S5_L_ and S6_T_ suggested in the closed state (recent work of Wisedchaisri *et al*. on the bacterial channel Na_V_Ab^19^) may completely reconfigure in the open state (studies on NavMs^8,16,17^, and also the present study on Na_V_Sp1 and Na_V_1.4). Interestingly, S4-S5_L_ residues implicated in the interaction with S6_T_ in the Na_V_Ab closed state (highlighted in Supplemental Fig. 3) do not align with the S4-S5_L_ residues implicated in the interaction with S6_T_ in the Na_V_Ms open state, suggesting the aforementioned reconfiguration of the interaction between S4-S5 linker and S6_T_. In addition, effects may be moderate because the fast kinetics of Na_V_ channel do not give the S4-S5_L_ peptides enough time to outcompete the endogenous S4-S5_L_ from interacting with S6_T_.

Regarding peptides effect on hNa_V_1.4, we took the option to co-express the pore-forming subunit with the auxiliary subunit hNa_V_ß1 to be closer to physiological conditions. Because of the presence of hNa_V_ß1, we cannot exclude that peptides effects on hNa_V_1.4 are mediated by this subunit. However, (i) the similitude of the peptide effects independently of the domain (I, II or III), (ii) the absence of domain IV peptide effect and (iii) the similitude of hNa_V_1.4 results to those observed on Na_V_Sp1 channel, lacking auxiliary subunit, suggest that the effect is rather specific to the pore-forming subunit.

From the present and previous studies, it seems that the coupling between voltage sensor movement and pore gating falls into two categories:

- the mechanical lever model: an obligatory coupling in which S4 resting state directly translates into S6 gate closed state. This obligatory coupling may be described as a simple mechanical work. At rest, S4-S5 linker helices compress the S6 helices and maintain the pore closed. Upon membrane depolarization, an outward displacement of S4 relieves the compression, allowing pore opening^10^. This model of electromechanical coupling is likely for Shaker-like channels in which open probability is very close to zero at hyperpolarizing potentials^22^;
- the ligand/receptor model: the obligatory coupling cannot hold if the S6 gate is able to open, even if S4 segments are in the resting state, as shown for hK_V_11.1 and hK_V_7.1 channels^23,24,35^. In this case, coupling is allosteric rather than obligatory: in these K_V_ channels, S4 resting state favors rather than forces channel closing. This allosteric coupling is realized through a ligand/receptor mechanism between S4-S5_L_ and S6_T_ in hK_V_7.1, hK_V_11.1 and hK_V_10.2 channels^25,26,29^. At rest, S4-S5_L_ binds to S6_T_ and stabilizes the channel in a closed state. If S4-S5_L_ affinity to S6_T_ is low enough, S4-S5_L_ and S6_T_ interaction is not permanent in S4 resting state, allowing transient pore opening. This is consistent with mutations in S4-S5_L_ and S6_T_ increasing the fraction of constitutively active current^9,23,35,36^.

Together, structural studies and the present study suggest that Na_V_ channels combine both mechanical lever (obligatory) and ligand/receptor (allosteric) models: (i) Structural data of various Na_V_ channel point to an electromechanical (obligatory) coupling model in which the four S4-S5 linkers are organized in a constriction ring that forces the channel gate to close when membrane is polarized. Upon membrane depolarization, movement of the S4 induces a lateral dilation of the S4–S5 linker, leading to a rotation and bending of the pore-lining S6 segments, which ultimately open the activation gate^10,12,15,20,21,37^. (ii) The present study, associated with studies on Na_V_Ms^8,16,17^, suggests an allosteric coupling: when S4 segments are in the activated conformation, S4-S5 linkers bind to S6_T_ and stabilize the channel in the open state. Various Na_V_1.4 structures, all showing S4 in the activated state but showing the S6 gate either closed^15^ or open^11,21^, are consistent with this allosteric mechanism in which S4, when activated, is not strongly coupled to S6_T_, but rather develop interactions with S6_T_ favoring the channel open state.

Many mutations of the hNa_V_1.4-encoding gene, *SCN4A*, are linked to muscular channelopathies^38^. The role of the S4-S5 linker as a modulator of the channel open state is consistent with the identification of several mutations in the area corresponding to the activating peptides: L689F, I692M, I693T in domain II and V1149L, A1152D and P1158S in domain III (underlined in Fig. 1B). Noteworthy, mutations in and around these sites (L689F, I692M, N1151C, A1152C, A1160C, P1158S) impair activation kinetics^39–45^.

Finally, mimicking peptides engineered for hK_V_7.1, hK_V_11.1, hK_V_10.2 and now hNa_V_1.4 may lead to a new therapeutic strategy for cardiac, neuronal and muscular channelopathies^46^.

## Methods

Similar methods have been used in previous studies^25,26,29^.

### Plasmid constructs

For Na_V_Sp1 and hNa_V_1.4, S4-S5_L_ plasmids were designed from the alignment with hK_V_11.1 and hK_V_7.1 S4-S5_L_ peptides. First, multiple-sequence alignment was realized with Cobalt^47^. This program aligned the predicted/observed S4 and S5 transmembrane domains of hK_V_11.1 (Uniprot Q12809), hK_V_7.1 (P51787), Na_V_Sp1^14,48^, and the four domains of hNa_V_1.4 (Uniprot P35499). We designed a Na_V_Sp1 S4-S5_L_ peptide and also a Na_V_1.4 S4-S5_L_ peptide in each domain, based on the aligned most potent hK_V_7.1 and hK_V_11.1 S4-S5_L_ peptides, as shown in Fig. 1B^25,26^. Since a peptide shifted by three amino acids toward the C-terminus also inhibited hK_V_7.1 (L251-L266), we also selected the corresponding peptide and also the next one in case of slight differences in binding sites. Thus, for Na_V_Sp1 and each hNa_V_1.4 domain, three different S4-S5_L_ plasmids were designed. All the peptides had the same length (16 amino-acids). Names of the peptides were given according to their position along the sequence: S4-S5_L_ (−3), S4-S5_L_ (0), and S4-S5_L_ (+3) (Fig. 1B). Two peptides, corresponding to hKv11.1 S4-S5 linker (A536-F551) and hKv11.1 C-terminus of S6 (I663-T675) were used as two negative controls. Oligonucleotides encoding hNa_V_1.4 and Na_V_Sp1 peptides were synthesized by TOP Gene Technologies and contained a XhoI restriction enzyme, a methionine (ATG) for translation initiation, and a glycine (GGA) to protect the ribosome binding site during translation and the nascent peptide against proteolytic degradation^49^. A BamHI restriction enzyme site was synthesized at the 3′ end immediately following the translational stop codon (TGA). These oligonucleotides were then cloned into pIRES2-EGFP (Clontech) and sequenced. Mutant Na_V_Sp1 were generated by using the QuikChange site-directed mutagenesis kit (Stratagene).

### Cell culture and transfection

The African green monkey kidney-derived cell line, COS-7, was obtained from the American Type Culture Collection (CRL-1651) and cultured in Dulbecco’s modified Eagle’s medium (GIBCO) supplemented with 10% fetal calf serum and antibiotics (100 IU/ml penicillin and 100 µg/ml streptomycin) at 5% CO_2_ and 95% air, maintained at 37 °C in a humidified incubator. Cells were transfected in 35-mm Petri dishes when the culture reached 50–60% confluence, with DNA (2 µg total DNA) complexed with FuGENE-6 (Roche Molecular Biochemical) according to the standard protocol recommended by the manufacturer. For hNa_V_1.4 experiments, COS-7 cells were co-transfected with 0.4 µg pRC-hNa_V_1.4, 0.4 µg pRC-hNa_V_ß1 (kind gifts of AL George, Northwestern University, Feinberg School of Medicine) and 1.2 µg pIRES2-EGFP plasmid (Clontech) encoding control or test peptides. For the experiments with the combination of peptides, COS-7 cells were co-transfected with 0.4 µg pRC-hNa_V_1.4, 0.4 µg pRC-hNa_V_ß1 and 0.6 µg of each of the two peptides encoding plasmid. For Na_V_Sp1 experiments, COS-7 cells were co-transfected with 0.8 µg pIRES2-EGFP-Na_V_Sp1 in which EGFP was removed and 1.2 µg pIRES2-EGFP plasmid encoding a control or a test peptide. Plasmid quantities were optimized to maximize the quantity of peptides, as assessed by the amount of fluorescence, and to keep current amplitudes in such a range that (i) undetectable currents were rare, and (ii) large currents inducing incorrect voltage-clamp were also rare. In pIRES2-EGFP plasmids, the second cassette (EGFP) is less expressed than the first cassette, guaranteeing high level of peptide expression in fluorescent cells^25^. For experiments with mutant Na_V_Sp1, COS-7 cells were transfected with 2 µg pIRES2-EGFP-Na_V_Sp1. Cells were re-plated onto 35-mm Petri dishes the day after transfection for patch-clamp experiments.

### Electrophysiology

The day after splitting, COS-7 cells were mounted on the stage of an inverted microscope and constantly perfused by a Tyrode solution (cf. below) at a rate of 1–3 ml/min. The bath temperature was maintained at 22.0 ± 2.0 °C. Stimulation and data recording were performed with pClamp 10, an A/D converter (Digidata 1440A) and an Axopatch 200B amplifier (all Molecular Devices). Patch pipettes (tip resistance: 1.5–2.2 MOhms) were pulled from soda lime glass capillaries (Kimble-Chase) and coated with dental wax to decrease capacitive currents. Currents were acquired in the whole-cell configuration, filtered at 10 kHz and recorded at a sampling rate of 20 kHz. Series resistance were compensated to 70–80%. To measure the Na_V_Sp1 currents, from a holding potential of −90 mV, the membrane was depolarized to 30 mV for 300 ms every 5 s. Na_V_Sp1 current was calculated after leak subtraction. To generate the activation curve, from a holding potential of −90 mV, the membrane was depolarized to values between −60 mV and +80 mV (+10 mV increment) for 300 ms, every 5 s. To measure the Na_V_1.4 current density after complete recovery from inactivation at −100 mV, a single step protocol was used to monitor current increase during recovery. From a holding potential of −100 mV, membrane was depolarized to 0 mV for 30 ms every 2 s. To generate the activation curve, from a holding potential of −100 mV, the membrane was depolarized to values between −70 mV and +50 mV (+5 mV increment) for 30 ms, every 2 s. As for Na_V_Sp1, Na_V_1.4 current was calculated after leak subtraction. To generate the inactivation curve, from a holding potential of −100 mV, membrane was depolarized to values between −110 mV and +25 mv (+5 mV increment) for 500 ms, followed by a 20-ms test pulse to 0 mV, every 4 s. Activation and inactivation curves were fitted by Boltzmann equations. G/V curves are obtained as follows: G_Na_ was calculated from G_Na_ = I_Na_/(V − V_rev_), where I_Na_ is the peak sodium current, V is the membrane potential and V_rev_ is the reversal potential estimated for each cell by linear regression of the linear rectification of I/V curve, when channels are fully activated. GV curves were subsequently obtained by dividing at each potential the peak current by the corresponding value of the linear regression curve.

### Solutions

The cells were continuously superfused with a HEPES-buffered Tyrode solution containing (in mmol/L): NaCl 145, KCl 4, MgCl_2_ 1, CaCl_2_ 1, HEPES 5, glucose 5, pH adjusted to 7.4 with NaOH. Patch pipettes were filled with the following solution (in mmol/L): KCl 90, Kgluconate 45, NaCl 10, HEPES 10, pH adjusted to 7.2 with KOH.

### Cell surface biotinylation assays

Surface biotinylation of transfected COS-7 cells (same condition as for patch-clamp experiments) was completed as described previously^50^. Briefly, cells were incubated with 0.5 mg/ml EZ-Link Sulfo-NHS-SS-Biotin (Pierce) in PBS, pH 7.4, for 30 min on ice. The biotinylation reaction was quenched with Tris-saline solution (10 mmol/L Tris, pH 7.4, 120 mmol/L NaCl), and detergent-soluble cell lysates were prepared. Biotinylated cell surface proteins were affinity-purified using Streptavidin-conjugated agarose beads (Pierce), and analyzed by western blot as described previously^50^. Bands corresponding to hNa_V_1.4 were normalized to bands corresponding to TransR from the same sample. hNa_V_1.4 protein expression (total or biotinylated fraction) in cells co-transfected with test peptides is expressed relative to hNa_V_1.4 protein expression (total or biotinylated fraction) in cells co-transfected with Control 1 peptide-encoding plasmid. Antibodies used were anti-Na_V_PAN mouse monoclonal antibody (Sigma, S8809), mouse monoclonal antibody against the transferrin receptor (Invitrogen, 13-6890), and a mouse monoclonal antibody against GAPDH (Santa Cruz Biotechnology, sc-32233). Anti‐mouse horseradish peroxidase–conjugated secondary antibody was purchased from Santa Cruz Biotechnology.

### Statistics

All data are expressed as mean ± sem. Statistical differences between samples were determined using Student’s t-tests when data were normally distributed (biophysical parameters) and rank-sum tests (Mann Whitney test) when data were not normally distributed (current densities). A value of p < 0.05 versus both controls was considered significant.

## Supplementary information

Supplementary File.
