# Supplementary material for: Up-regulation of voltage-gated sodium channels by peptides mimicking S4-S5 linkers reveals a variation of the ligand-receptor mechanism
Source: Sci Rep. 2020 Apr 3;10:5852. doi: 10.1038/s41598-020-62615-6 (PMC7125111; doi:10.1038/s41598-020-62615-6)
Supplement: Supplementary file 1 — Supplementary File. [file 41598_2020_62615_MOESM1_ESM.pdf]

# Up-regulation of voltage-gated sodium channels by peptides mimicking S4-S5 linkers reveals a variation of the ligand-receptor mechanism

Olfat A. Malak [1,2,$], Fayal Abderemane-Ali [1,3,$], Yue Wei [1,4], Fabien C. Coyan [1], Gilyane Pontus [1], David Shaya[5], Céline Marionneau [1], Gildas Loussouarn [1]

1. *Université de Nantes, CNRS, INSERM, l'institut du thorax, F-44000 Nantes, France.*
2. *Present address: 8001 Redwood Blvd, Novato, California 9494, USA5*
3. *Present address: Cardiovascular Research Institute, University of California, San Francisco, California 941158-9001, USA*
4. *Present address: Department of Cardiology, Shanghai Ruijin Hospital, Shanghai Jiao Tong University School of Medicine, Shanghai, China*
5. *Cardiovascular Research Institute, University of California, San Francisco, California 941158-9001, USA*

[$] ***These authors contributed equally to this work.***

**Address correspondence to:**
Dr. Gildas Loussouarn
L'institut du thorax
INSERM UMR 1087 / CNRS UMR 6291
IRS-UN, 8 Quai Moncousu BP 70721
44007 Nantes cedex 1, France
Tel: +33 (0)2 2808 0150
Fax: +33 (0)2 2808 0130
E-mail: gildas.loussouarn@inserm.fr

Keywords: $Na_V$1.4 channel – $Na_V$Sp1 channel – voltage dependence – S4-S5 linker – S6 C-terminus

| | Control 1 | Control 2 | S4-S5$_L$ (-3) DI | S4-S5$_L$ (0) DI | S4-S5$_L$ (+3) DI |
|---|---|---|---|---|---|
| **Current densities (n)** | 45 | 40 | 47 | 20 | 40 |
| **at 30mV (pA/pF)** | - 12.3 ± 2.8 | - 12.0 ± 4.0 | - 16.9 ± 3.2 | - 17.2 ± 4.9 | -23.1 ± 6.2 |
| **P-values *vs* control 1** | -- | 0.46 | 0.049 | 0.16 | 0.09 |
| **P-values *vs* control 2** | 0.46 | -- | 0.03 | 0.12 | 0.06 |
| **Activation (n)** | 8 | 10 | 17 | 8 | 10 |
| **$V_{0.5}$ Act (mV)** | 30.4 ± 1.9 | 29.3 ± 1.8 | 23.5 ± 1.8 | 26.4 ± 2 | 25.9 ± 2.7 |
| **P-values *vs* control 1** | -- | 0.69 | 0.03 | 0.17 | 0.22 |
| **P-values *vs* control 2** | 0.69 | -- | 0.04 | 0.31 | 0.32 |
| **K Act (mV)** | 11.1 ± 0.7 | 11.4 ± 0.9 | 9.9 ± 0.4 | 10.4 ± 0.6 | 9.6 ± 0.5 |
| **P-values *vs* control 1** | -- | 0.79 | 0.11 | 0.09 | 0.34 |
| **P-values *vs* control 2** | 0.79 | -- | 0.39 | 0.08 | 0.09 |

**Supplemental Table 1: Effect of $Na_V$Sp1 S4-S5$_L$ mimicking peptides on $Na_V$Sp1 current density and activation curve.** Mean ± sem peak current densities recorded at +30 mV, in COS-7 cells co-transfected with $Na_V$Sp1 and the indicated peptide. Mean ± sem half-activation potential and slope of the activation curves, obtained using the activation protocol shown in Figure 2. Mann-Whitney tests for current densities and Student's t-tests for activation parameters ($V_{0.5}$ and K).

| | Control 1 | Control 2 | S4-S5$_L$ (-3) DI | S4-S5$_L$ (0) DI | S4-S5$_L$ (+3) DI | S4-S5$_L$ (-3) DII | S4-S5$_L$ (0) DII | S4-S5$_L$ (+3) DII | S4-S5$_L$ (-3) DIII | S4-S5$_L$ (0) DIII | S4-S5$_L$ (+3) DIII | S4-S5$_L$ (-3) DIV | S4-S5$_L$ (0) DIV | S4-S5$_L$ (+3) DIV |
|---|---|---|---|---|---|---|---|---|---|---|---|---|---|---|
| Current densities (n) | 119 | 110 | 18 | 24 | 21 | 20 | 25 | 22 | 20 | 21 | 25 | 31 | 19 | 22 |
| at 0mV (pA/pF) | -67.4 ± 6.3 | -60.5 ± 5.2 | -84.4 ± 18.1 | -70.9 ± 10.2 | -102.9 ± 16.9 | -93.1 ± 22.3 | -76.2 ± 16.6 | -99.1 ± 17.3 | -69.9 ± 16.9 | -90.1 ± 14.7 | -58.7 ± 8.9 | -80.4 ± 14.6 | -63.9 ± 9.4 | -97.9 ± 19.4 |
| P-values *vs* control 1 | -- | 0.38 | 0.21 | 0.16 | 0.01 | 0.13 | 0.42 | 0.03 | 0.41 | 0.04 | 0.47 | 0.28 | 0.26 | 0.06 |
| P-values *vs* control 2 | 0.38 | -- | 0.15 | 0.15 | 0.007 | 0.11 | 0.35 | 0.02 | 0.49 | 0.02 | 0.41 | 0.21 | 0.21 | 0.05 |
| Activation (n) | 58 | 49 | 6 | 12 | 10 | 9 | 5 | 11 | 9 | 13 | 12 | 17 | 13 | 10 |
| $V_{0.5}$ Act (mV) | -12.8 ± 0.6 | -13.3 ± 0.6 | -13.3 ± 1.6 | -10.7 ± 0.7 | -14.1 ± 1.5 | -12.4 ± 1.8 | -15.5 ± 2.4 | -13.7 ± 1.1 | 18.1 ± 2.3 | 12.4 ± 0.9 | -13.6 ± 1.9 | -13 ± 1.1 | -12.5 ± 1.4 | 12.2 ± 1.6 |
| P-values *vs* control 1 | -- | 0.59 | 0.81 | 0.15 | 0.41 | 0.83 | 0.25 | 0.53 | 0.005 | 0.79 | 0.62 | 0.84 | 0.88 | 0.76 |
| P-values *vs* control 2 | 0.59 | -- | 0.99 | 0.06 | 0.58 | 0.61 | 0.32 | 0.75 | 0.009 | 0.52 | 0.84 | 0.86 | 0.62 | 0.53 |
| K Act (mV) | 6.8 ± 0.1 | 6.7 ± 0.1 | 6.5 ± 0.4 | 6.9 ± 0.3 | 6.5 ± 0.1 | 6.5 ± 0.3 | 6.1 ± 0.4 | 6.6 ± 0.2 | 6.1 ± 0.3 | 6.5 ± 0.1 | 7.1 ± 0.3 | 6.8 ± 0.2 | 6.9 ± 0.2 | 6.4 ± 0.2 |
| P-values *vs* control 1 | -- | 0.69 | 0.43 | 0.67 | 0.38 | 0.34 | 0.13 | 0.6 | 0.049 | 0.27 | 0.37 | 0.90 | 0.64 | 0.22 |
| P-values *vs* control 2 | 0.69 | -- | 0.37 | 0.41 | 0.32 | 0.28 | 0.04 | 0.66 | 0.01 | 0.20 | 0.26 | 0.88 | 0.34 | 0.14 |
| Inactivation (n) | 28 | 22 | 11 | 12 | 10 | 12 | 9 | 13 | 9 | 12 | 13 | 14 | 14 | 11 |
| $V_{0.5}$ Inact (mV) | -60.3 ± 1.2 | -62.1 ± 1.3 | -59.3 ± 0.7 | -62.5 ± 1.9 | -57.7 ± 1.0 | -61.5 ± 2.0 | -61.3 ± 1.7 | -59.2 ± 0.9 | -59.1 ± 0.9 | -58.1 ± 0.9 | -61.7 ± 2.6 | -60.8 ± 1.4 | -61.2 ± 1.3 | -58.2 ± 1.2 |
| P-values *vs* control 1 | -- | 0.33 | 0.59 | 0.33 | 0.22 | 0.59 | 0.67 | 0.56 | 0.57 | 0.25 | 0.58 | 0.80 | 0.65 | 0.31 |
| P-values *vs* control 2 | 0.33 | -- | 0.16 | 0.86 | 0.048 | 0.81 | 0.74 | 0.14 | 0.18 | 0.05 | 0.89 | 0.53 | 0.66 | 0.07 |
| K Inact (mV) | 5.1 ± 0.2 | 5.2 ± 0.1 | 5.1 ± 0.3 | 5.5 ± 0.3 | 6 ± 0.2 | 5.4 ± 0.2 | 5.7 ± 0.4 | 5.9 ± 0.4 | 5.5 ± 0.5 | 5.7 ± 0.4 | 6.2 ± 0.4 | 5.7 ± 0.3 | 5.6 ± 0.2 | 5.3 ± 0.4 |
| P-values *vs* control 1 | -- | 0.73 | 0.87 | 0.24 | 0.03 | 0.38 | 0.16 | 0.08 | 0.37 | 0.17 | 0.02 | 0.15 | 0.16 | 0.63 |
| P-values *vs* control 2 | 0.73 | -- | 0.92 | 0.22 | 0.007 | 0.35 | 0.12 | 0.07 | 0.40 | 0.16 | 0.01 | 0.15 | 0.13 | 0.75 |

**Supplemental Table 2: Effect of $Na_V$1.4 S4-$S5_L$ mimicking peptides on $Na_V$1.4 current density and activation/inactivation curves.** Mean ± sem tail current densities recorded at 0 mV, in COS-7 cells co-transfected with h$Na_V$1.4, h$Na_V$ß1, and the indicated peptide. Half-activation/inactivation potentials and slopes of the activation/inactivation curves, obtained from tail currents, using the protocols shown in figures 5,6,7,8 and suppl. figures 6,7,8,9. Mann-Whitney tests for current densities and Student's t-tests for activation parameters ($V_{0.5}$ and K).

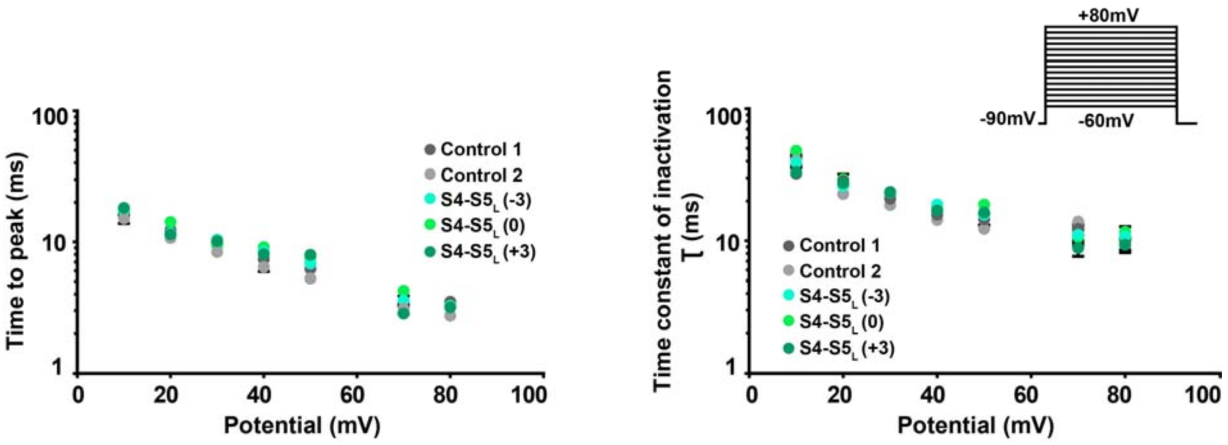


**Supplemental Figure 1:** Effect of $Na_V$Sp1 S4-S5$_L$ mimicking peptides on $Na_V$Sp1 activation and inactivation kinetics. Left: mean ± sem time to peak of the sodium current measured at various potentials. Right: mean ± sem $Na_V$Sp1 inactivation time constant, obtained from a monoexponential fit of the inactivating current (n=7-10).

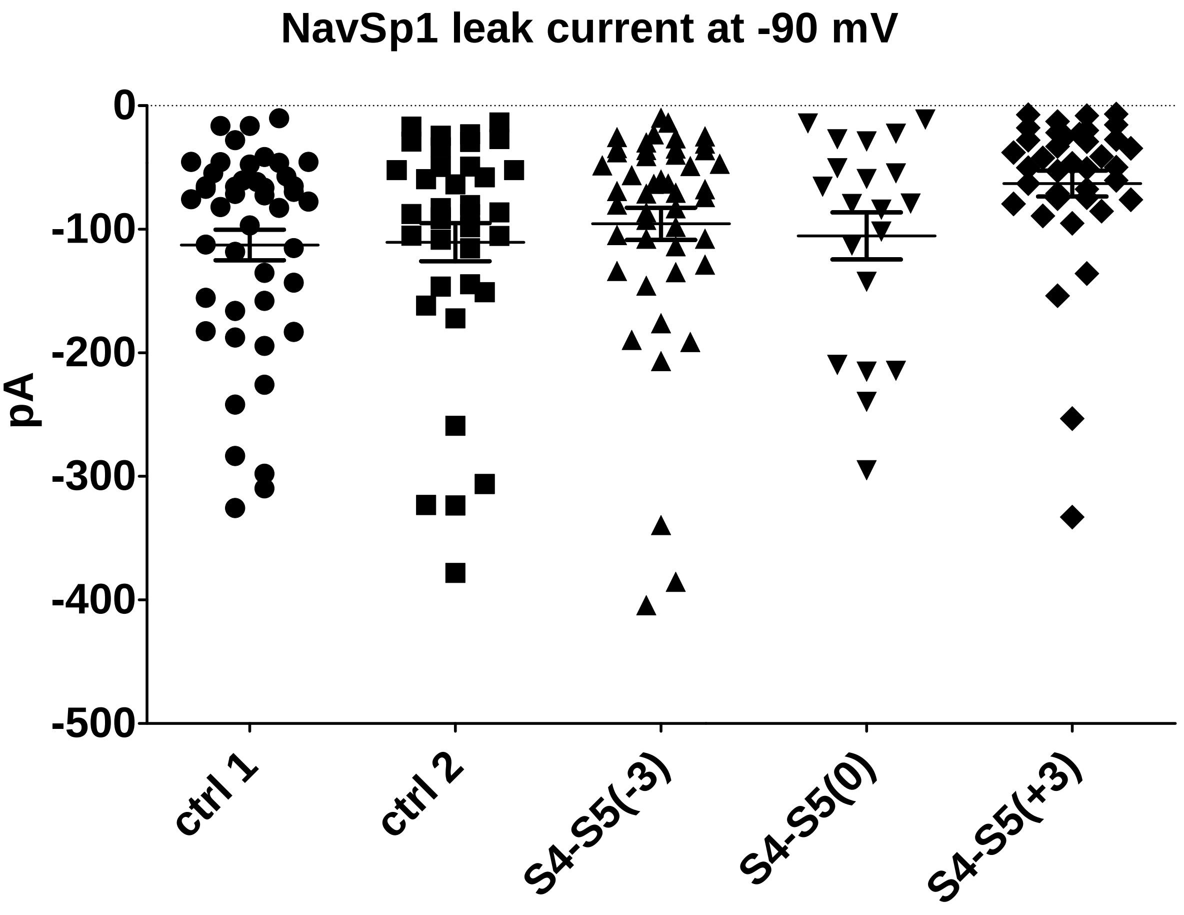


**Supplemental Figure 2:** Effect of $Na_VSp1$ S4-$S5_L$ mimicking peptides on $Na_VSp1$ current measured at -90 mV.

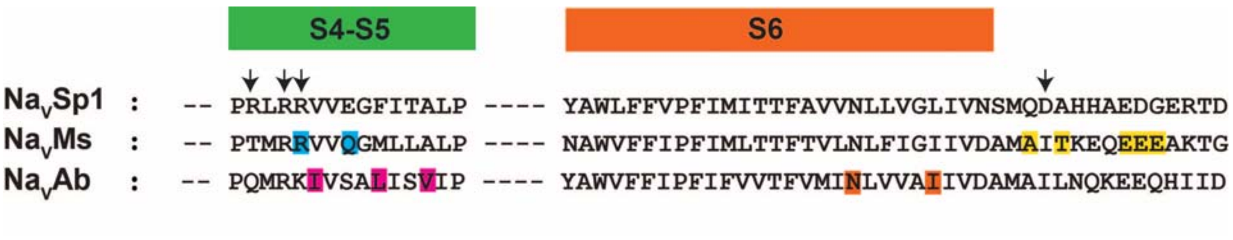


**Supplemental Figure 3.** Alignment of NavSp1, NavMs and NavAb channels indicating the interacting S4-S5L (blue) and S6T (yellow) amino-acids in the NavMs open state and the interacting S4-S5L (magenta) and S6T (orange) amino-acids in the NavAb closed state. Figure extracted from ref. 16 and modified. Arrows indicate residues that were selected for mutagenesis experiments presented in figures 3 and 4.

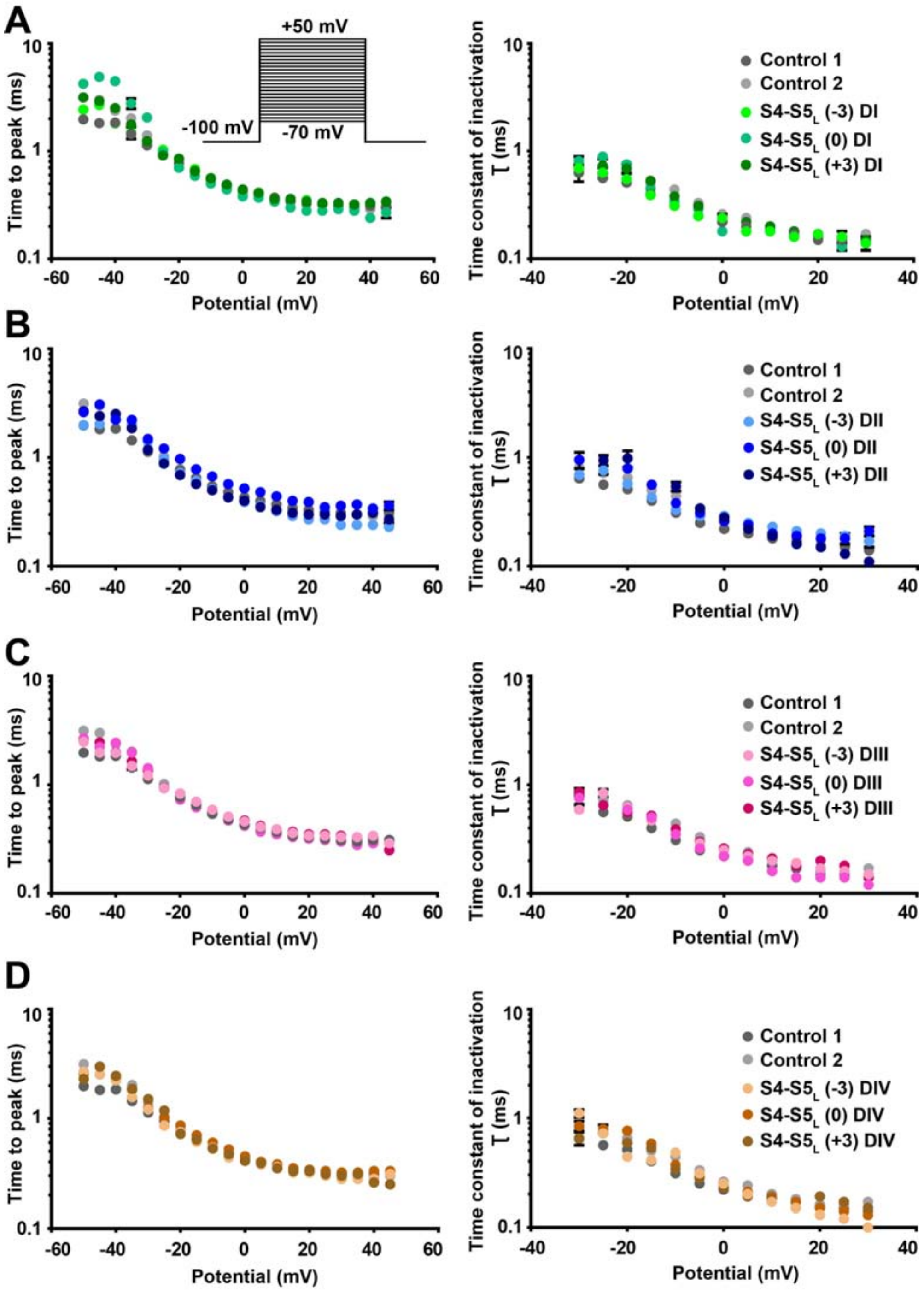


**Supplemental Figure 4:** Effect of domain I, II, III and IV (A, B, C and D) S4-S5$_L$ mimicking peptides on Na$_V$1.4 activation and inactivation kinetics. Left: mean ± sem time to peak of the sodium current measured at various potentials. Right: mean ± sem Na$_V$1.4 inactivation time constant, obtained from a monoexponential fit of the inactivating current (n=6-43).

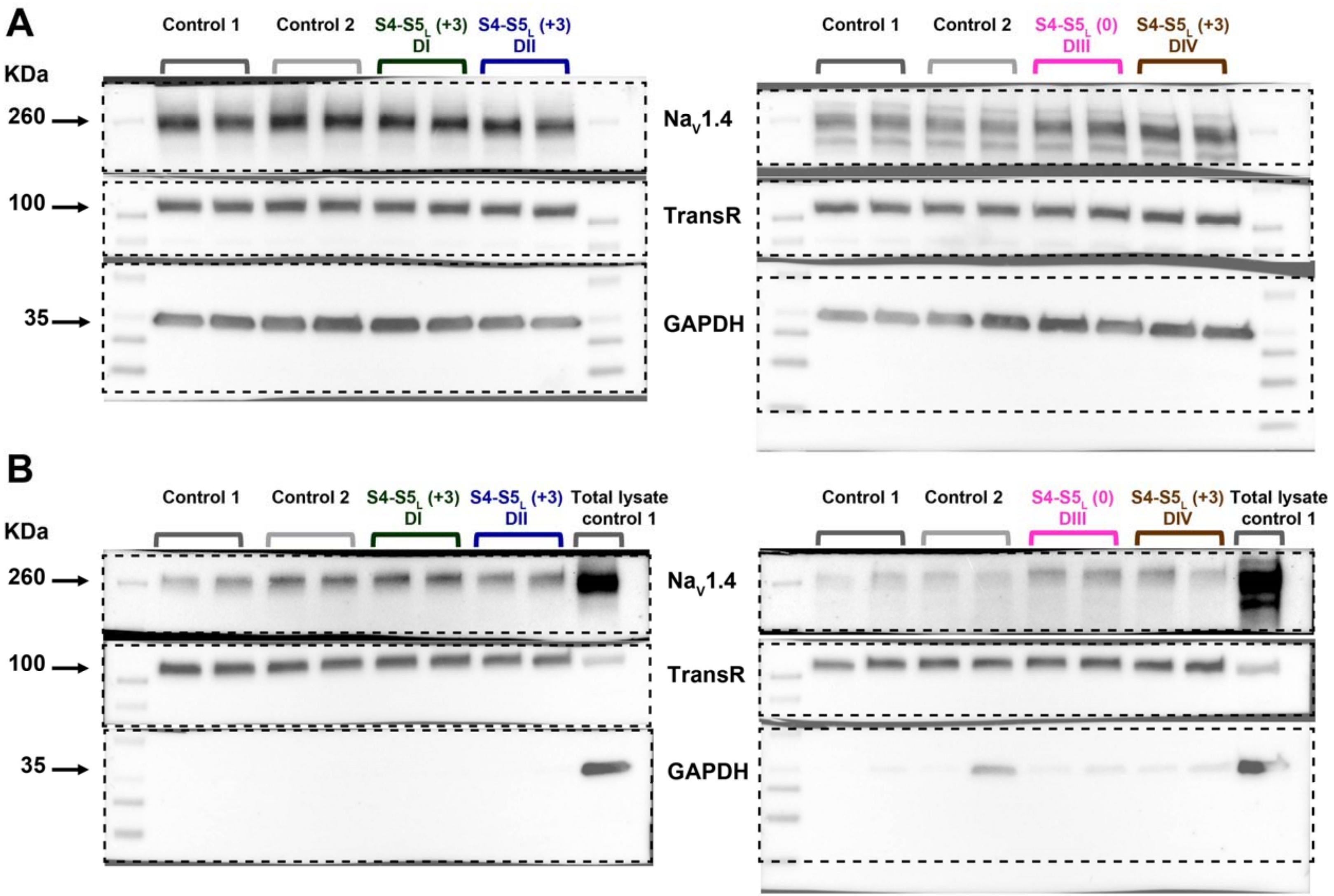


**Supplemental Figure 5: Original Western blot panels corresponding to Figure 9.** Nav1.4, TransR and GAPDH proteins were detected on the same corresponding membranes. The membranes were cut in three pieces directly after the transfer. The boxes represent the bands shown in figure 9.

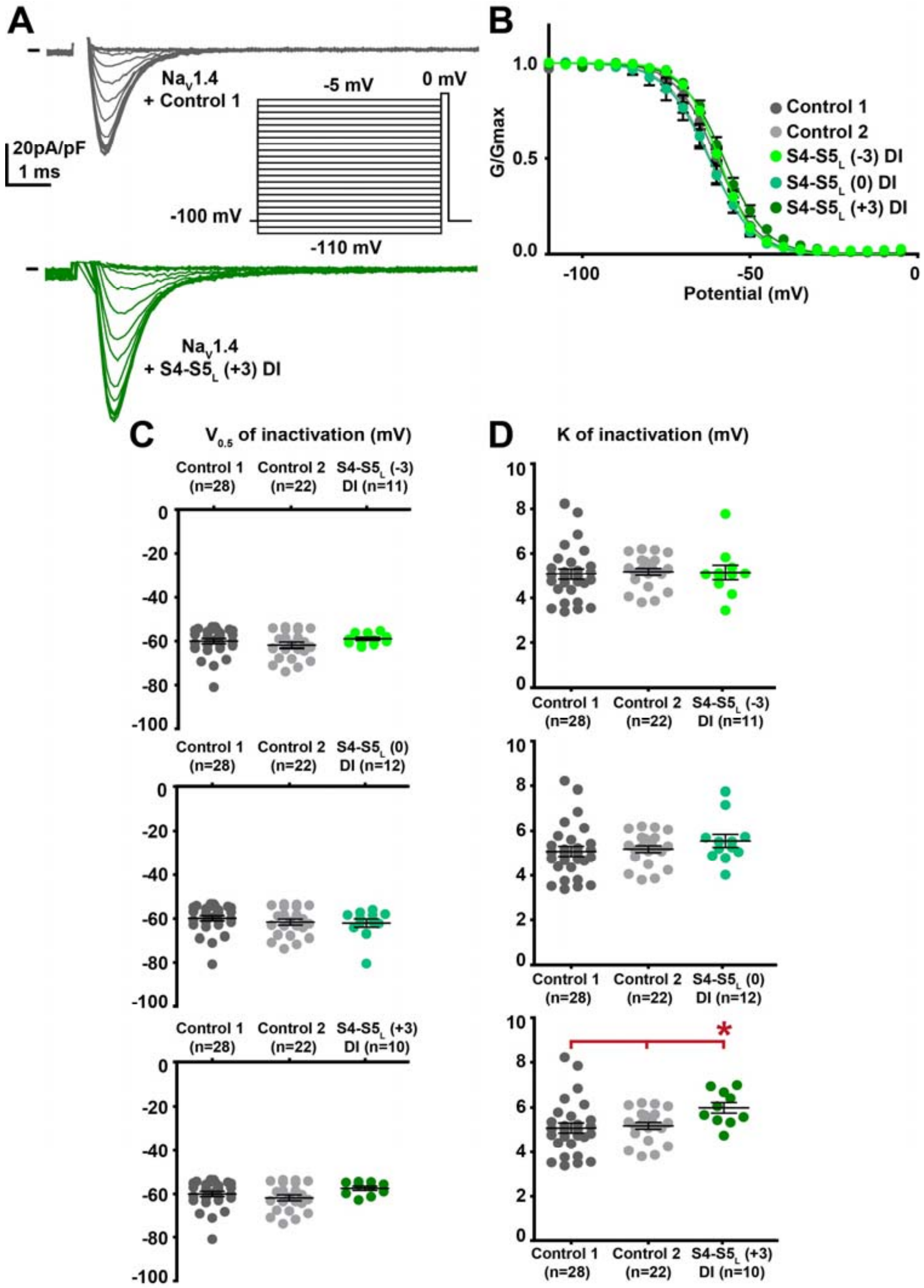


**Supplemental Figure 6. Effect of S4-S5$_L$ mimicking peptides of domain I on Na$_V$1.4 inactivation curve. A:** representative, superimposed recordings of the Na$_V$1.4 current in presence or absence of domain I S4-S5$_L$(+3). **Inset:** voltage protocol used (pre-pulse: 500 msec, test-pulse 20 msec to 0 mV, one sweep every 4 s). **B:** Steady-state channel availability curves for Nav1.4 channels. Data are mean normalized peak current (I/Imax) measured at 0 mV versus pre-pulse voltage. **C & D:** Mean ± sem of Na$_V$1.4 half-inactivation potential ($V_{0.5}$; **C)** and inactivation slope (K; **D)**, in COS-7 cells co-transfected with Na$_V$1.4, Na$_V$ß1, and the indicated peptide. *, $p < 0.05$ vs. both controls.

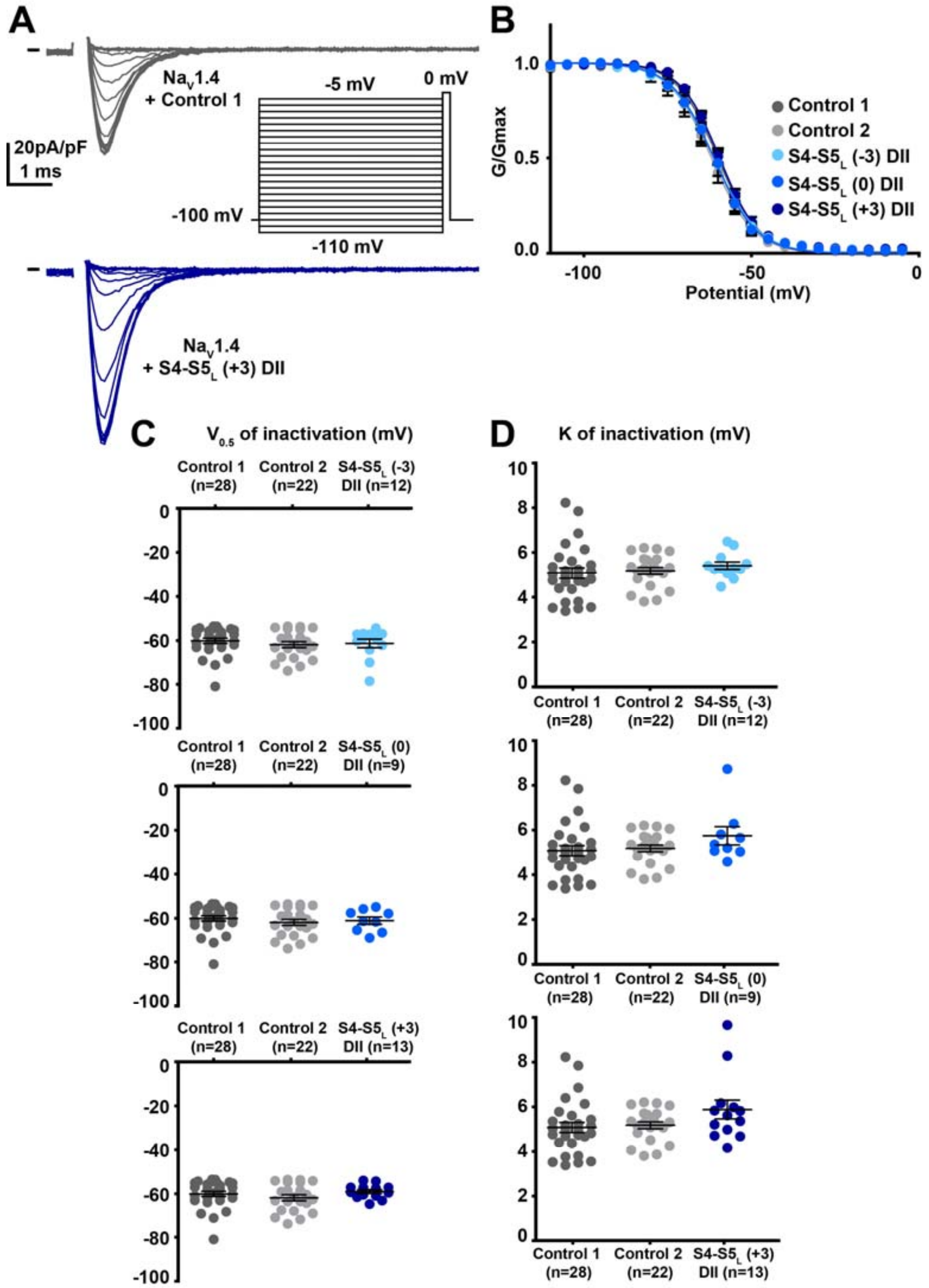


**Supplemental Figure 7. Effect of S4-S5$_L$ mimicking peptides of domain II on Na$_V$1.4 inactivation curve. A:** representative, superimposed recordings of the Na$_V$1.4 current in presence or absence of domain II S4-S5$_L$(+3). **Inset:** voltage protocol used (pre-pulse: 500 msec, test-pulse 20 msec to 0 mV, one sweep every 4 s). **B:** Steady-state channel availability curves for Nav1.4 channels. Data are mean normalized peak current (I/Imax) measured at 0 mV versus pre-pulse voltage. **C & D:** Mean ± sem of Na$_V$1.4 half-inactivation potential (V$_{0.5}$; **C)** and inactivation slope (K; **D)**, in COS-7 cells co-transfected with Na$_V$1.4, Na$_V$ß1, and the indicated peptide.

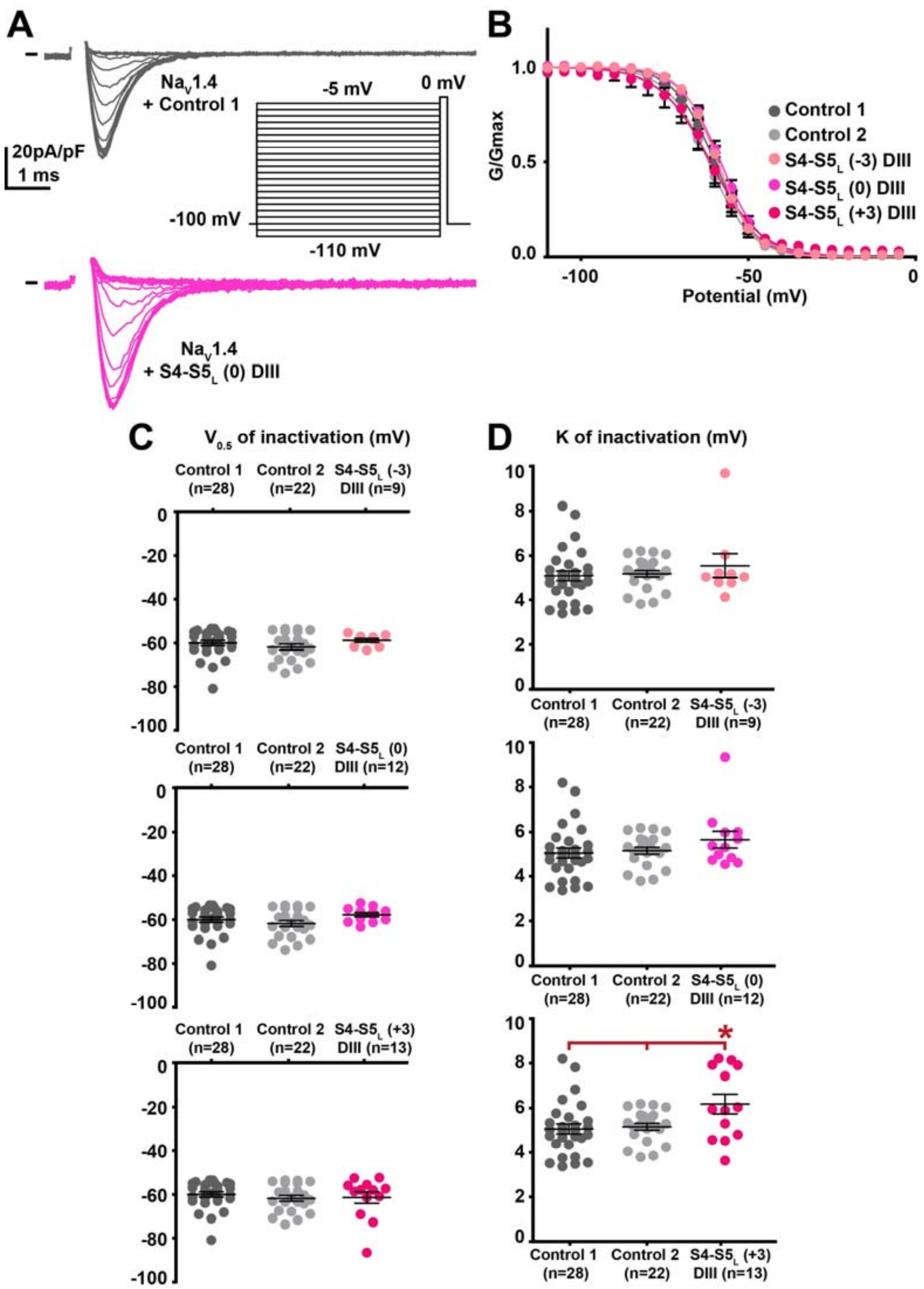


**Supplemental Figure 8. Effect of S4-S5$_L$ mimicking peptides of domain III on Na$_V$1.4 inactivation curve. A:** representative, superimposed recordings of the Na$_V$1.4 current in presence or absence of domain III S4-S5$_L$(0). **Inset:** voltage protocol used (pre-pulse: 500 msec, test-pulse 20 msec to 0 mV, one sweep every 4 s). **B:** Steady-state channel availability curves for Nav1.4 channels. Data are mean normalized peak current (I/Imax) measured at 0 mV versus pre-pulse voltage. **C & D:** Mean ± sem of Na$_V$1.4 half-inactivation potential (V$_{0.5}$; **C)** and inactivation slope (K; **D)**, in COS-7 cells co-transfected with Na$_V$1.4, Na$_V$ß1, and the indicated peptide. *, $p < 0.05$ vs. both controls.

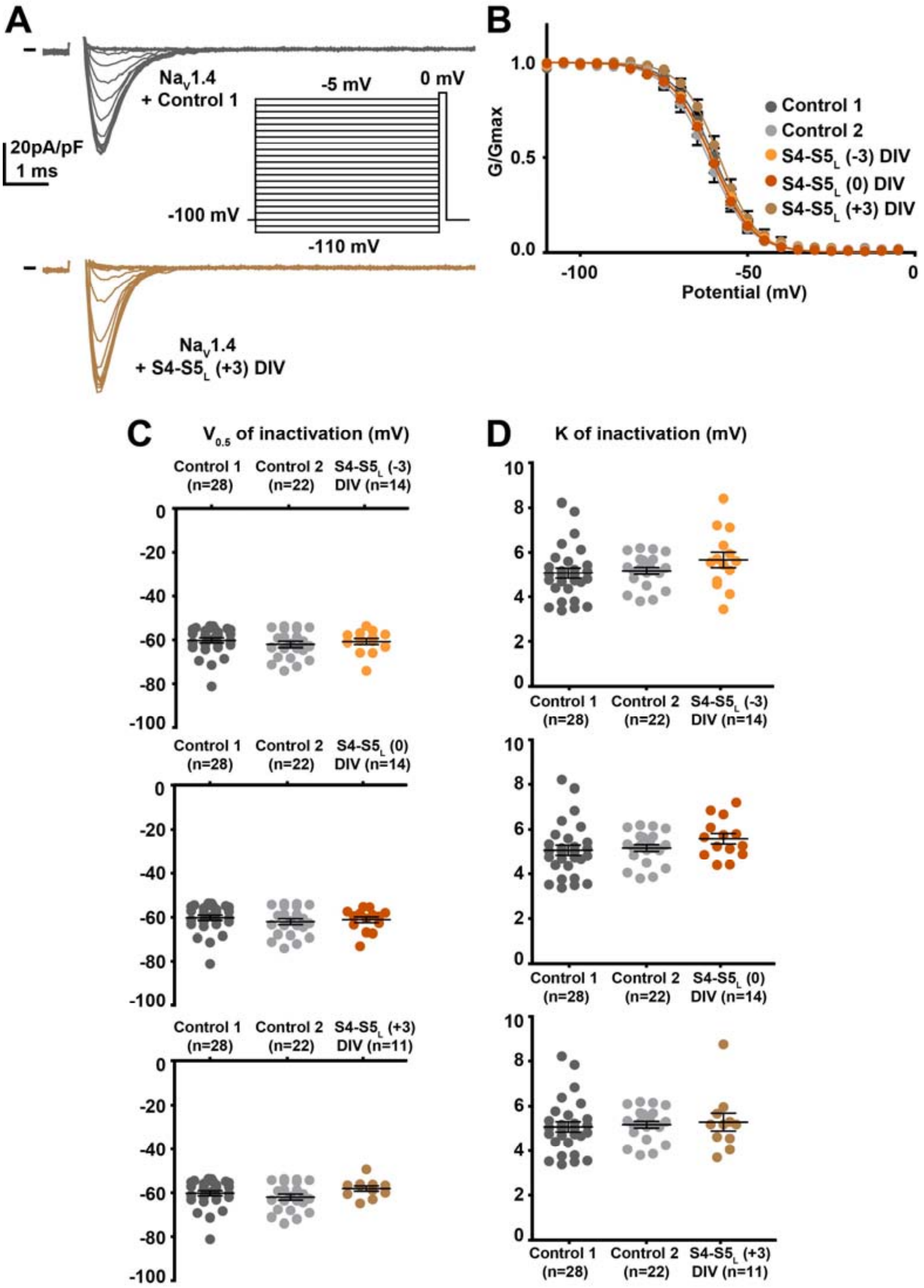


**Supplemental Figure 9. Effect of S4-S5$_L$ mimicking peptides of domain IV on Na$_V$1.4 inactivation curve. A:** representative, superimposed recordings of the Na$_V$1.4 current in presence or absence of domain IV S4-S5$_L$(+3). **Inset:** voltage protocol used (pre-pulse: 500 msec, test-pulse 20 msec to 0 mV, one sweep every 4 s). **B:** Steady-state channel availability curves for Nav1.4 channels. Data are mean normalized peak current (I/Imax) measured at 0 mV versus pre-pulse voltage. **C & D:** Mean ± sem of Na$_V$1.4 half-inactivation potential (V$_{0.5}$; **C)** and inactivation slope (K; **D)**, in COS-7 cells co-transfected with Na$_V$1.4, Na$_V$ß1, and the indicated peptide.

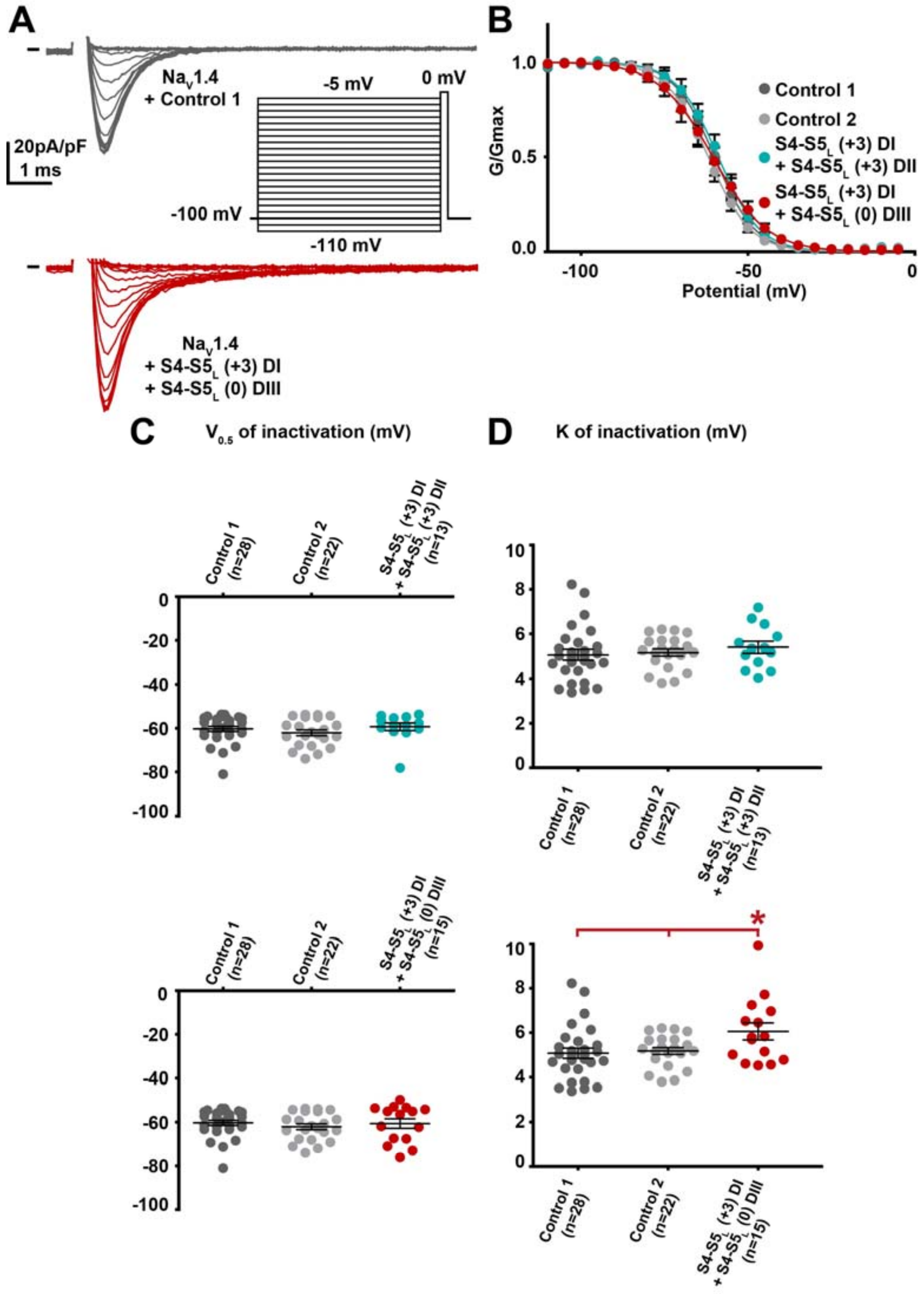


**Supplemental Figure 10. Effect of the combination of two activating S4-S5$_L$ mimicking peptides on Na$_V$1.4 inactivation curve. A:** representative, superimposed recordings of the Na$_V$1.4 current in presence or absence of the indicated peptides. **Inset:** voltage protocol used (pre-pulse: 500 msec, test-pulse 20 msec to 0 mV, one sweep every 4 s). **B:** Steady-state channel availability curves for Nav1.4 channels. Data are mean normalized peak current (I/Imax) measured at 0 mV versus pre-pulse voltage. **C & D:** Mean ± sem of Na$_V$1.4 half-inactivation potential ($V_{0.5}$; **C)** and inactivation slope (K; **D)**, in COS-7 cells co-transfected with Na$_V$1.4, Na$_V$ß1, and the indicated peptides. *, $p < 0.05$ vs. both controls.
